# The role of parenthood in worry about overheating in homes in the UK and the US and implications for energy use: An online survey study

**DOI:** 10.1371/journal.pone.0277286

**Published:** 2022-12-01

**Authors:** Gesche M. Huebner

**Affiliations:** University College London, Bartlett School of Environment, Energy and Resources, London, United Kingdom; Southwest Jiaotong University, CHINA

## Abstract

Climate change brings an increase in temperatures and a higher frequency of heatwaves. Both have been linked to a rise in suicide rates and violent crime on a population level. However, little is known about the link between mental health and ambient temperatures on an individual level and for particular subgroups. Overheating poses health risks to children and disturbs sleep; leading to the expectation that parents are more worried about their homes getting too hot than non-parents. Two online survey studies (N = 1000 each) were conducted in the UK and the US to understand to what extent parents and an age-matched comparison group without children are worried about overheating and how they differ in their mitigation actions. Findings did not support the main hypotheses around greater overheating worry amongst parents in general, mothers or those with very young children. However, parents indicated a greater likelihood to upgrade or install air-conditioning (US) and to get electric fans (UK). Parents in the UK indicated using more mitigation options to cope with overheating than non-parents. Parents in the US, whilst not reporting doing more actions, were more likely to use air-conditioning to deal with overheating than non-parents. Finally, those parents who mentioned health impacts on children as a reason for worry about overheating, were more concerned about overheating than parents who had other reasons than children’s health as a reason for being concerned about overheating. In summary, parental status might have implications for cooling energy use and concern for children’s health might increase overheating worry; however, many open questions remain.

## Introduction

Climate change has been identified as the greatest global health threat of current times but most research has focused on the physical health consequences of climate change [1] whereas the mental health consequences are generally an understudied area [2]. The financial implications of worsened mental health are substantial: Poor mental health was estimated to cost the world economy approximately $2.5 trillion per year in poor health and reduced productivity in 2010, with a projected rise to $6 trillion by 2030 [3], indicating that mental health must be a priority area.

One of the most prominent consequences of climate change will be an increase in ambient temperatures and heatwaves. Evidence has linked them to an increase in suicide rates [4–6], violent crime [7–9], and mental distress [10–12], largely using population based data. However, comparatively little is known about an association between mental health outcomes and high temperatures on an individual level and under particular consideration of overheating in homes as opposed to outside temperatures. Summertime overheating has been reported in many countries with low prevalence of air-conditioning, such as Germany and France [13]. Lomas et al. estimated that 19% of bedrooms and 15% of living rooms in England experienced overheating in 2018 [14]. There is no unified definition of overheating with both static criteria and adaptive criteria being used. In the former case a fixed temperature and exposure duration is used to define overheating whereas in the latter case the still acceptable indoor temperature increases and decreases in line with the outdoor temperature. The work presented here looks at overheating from a subjective point and respondents were simply asked if their home gets too hot; hence, a detailed discussion of overheating criteria is not relevant here but see e.g. [15,16].

Here, a comparison between overheating worry in parents and an age-matched comparison group without children is made. Younger people factor climate change concerns into their reproductive choices to some extent [17]. Whilst evidence does not suggest that having children is associated with an increase in environmentalism [18,19], it is plausible that parents are more concerned about the personal consequences of climate change given the potential negative impacts on their children. Sudden Infant Death Syndrome has been linked to higher ambient temperatures in multiple [20–22] though not all studies [23]. Sleep is more disturbed for babies sleeping in warmer rooms [24]. High temperatures are a greater health risk to children than adults because of physiologically reasons such as a faster breathing rate and a difference in skin surface area [25,26]. Some of the risks resulting from heat waves are renal disease, respiratory disease, electrolyte imbalance and fever [27] and heatwaves have been linked to an increase in emergency department visits [28]. Parents generally have high levels of concern for their children [29] and in particular mothers around childhood illness [30]. Women also traditionally take on the larger share of parental leave and childcare duties and hence do more of the ‘emotional labour’ within families [31], and 13% of women experience a mental disorder postnatally [32].

In summary, it is plausible to assume that overheating of one’s home causes concerns in parents, especially mothers. Yet, how parents might respond to overheating risks is understudied. One study has assessed how parents deal with overheating based on a qualitative data collected in Australia and found that parents used a wide range of coping behaviours for dealing with hot weather and also showed that official guidance is contradictory [33]. Parental wellbeing and concern was not assessed.

Instead of using diagnosed mental health conditions, here, this study focuses on worry about overheating and its implications for energy use. The comparatively low incidence of mental illnesses would mandate a very large sample size, again likely leading to an analysis focused on population-level estimates instead of linking individual-level variables (parental status) to individual-level outcome measures (overheating worry and mitigation actions).

Understanding the impacts of overheating on parents and non-parents will shed further light on the understudied research field of mental health and wellbeing impacts of climate change. From an energy point of view, coping strategies such as using fans or cooling devices will increase energy demand. Air-conditioning use is projected to rise significantly [34] and having a new-born may become an additional critical trigger for the uptake of air conditioning.

Two online studies in the US and the UK addressed the following research questions:

1. Are parents, in particular mothers and parents of young children, more worried about overheating in homes than an age-matched control group without children?
2. Are parents more likely to install cooling devices in the future and use more mitigation options against overheating than those without children?

Analysis is carried out separately for the UK and the US. The UK is historically a heating-dominated country with a low prevalence of air-conditioning; however, it is experiencing increasingly hotter summers and air-conditioning uptake is expected to increase. The US already has a high prevalence of air-conditioning and hence an effective tool against home overheating which could impact results [35]. Analysis was preregistered [36].

## Results

### Description of the sample

**Fig 1:**
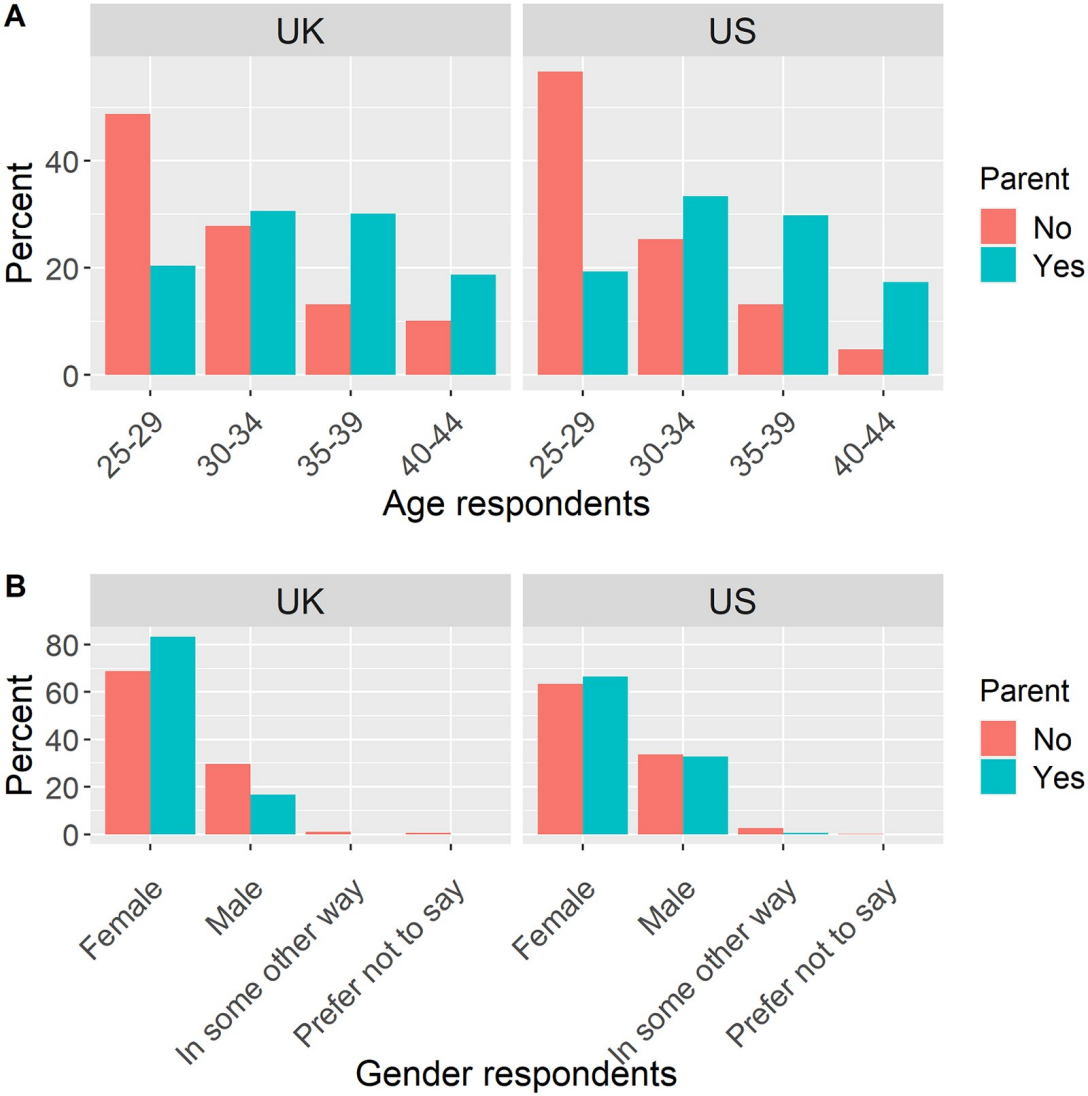
Age and gender split up by parental status in the UK and the US.

Following data cleaning (see Methods), the remaining sample size for the UK was N = 955 of which 48.5% were parents (N = 463) and hence 51.5% non-parents (N = 492) and for the US N = 950 of which 47.3% were parents (N = 449) and hence 52.7% non-parents (N = 501). Fig 1 shows the age and gender characteristics of both samples split up by parental status. The youngest age group was overrepresented in the non-parents sample in both US and UK and in general, the majority of respondents were female.

For information on work status, tenure and dwelling type, please see S1 Table.

### Overheating worry in parents and non-parents

Overheating worry was measured with six items on a four-point scale with low values reflecting low overheating worry and high values high overheating worry. Mean overheating worry was M = 1.94 (SD = 0.81) in the UK and M = 2.13 (SD = 0.88) in the US, i.e. relatively low levels of overheating worry. This difference was significant, i.e. mean overheating worry was higher in the US than the UK, *t*(1904) = 4.93, *p* < .001.

#### Hypothesis 1

Overheating worry was not significantly higher in parents (M = 1.97) than non-parents (M = 1.92) in the UK [*t*(953) = -0.97, *p* = .332)].In the US it was significantly lower in parents (M = 2.07) than in non-parents (M = 2.19), *t*(948) = 2.07, *p* = .0391. For the UK, the equivalence test was significant (for details, see S1 Appendix). The equivalence test and the null-hypothesis test combined indicated that the observed effect is statistically not different from zero and statistically equivalent to zero.

Using a Wilcoxon-Rank-Sum test, given the non-normality of the mean overheating worry, confirmed the finding of no difference in the UK and lower overheating worry in parents than non-parents in the US (W = 121965, *p* = .024).

Hence, hypothesis 1 was not confirmed, parents did not have higher overheating worry. For the US, overheating worry was higher in non-parents.

#### Hypothesis 2

Hypothesis 2 tested if mothers (N_UK_ = 387 [40.9%], N_US_ = 299 [32.0%)) had higher overheating worry than fathers (N_UK_ = 76 [8.0%], N_US_ = 147[15.8%]), women without children (N_UK_ = 338 [35.7%], N_US_ = 318 [34.1%]) or men without children (N_UK_ = 146 [15.4%], N_US_ = 169 [18.1%]). Respondents who had either not indicated their gender or chosen an ‘other’ category were excluded as their numbers were too low to form a category (see Fig 1). Table 1 shows the regression results for the UK sample (a) and the US sample (b).

**Table 1:** Relating overheating worry to gender and parental status. a) Regression results UK sample (mother as reference category). b) Regression results US sample (mother as reference category).

	**Overheating worry (UK)**		
*Predictors*	*Estimates*	*CI*	*p*
(Intercept)	1.97	1.89–2.05	**<0.001**
Mother	*Reference*		
Father	0.00	-0.20–0.20	0.995
Woman, no child	-0.02	-0.14–0.10	0.739
Man, no child	-0.13	-0.29–0.02	0.097
Observations	947		
R^2^ / R^2^ adjusted	0.003 / -0.000		
	**Overheating worry (US)**		
*Predictors*	*Estimates*	*CI*	*p*
(Intercept)	2.06	1.96–2.16	**<0.001**
Mother	*Reference*		
Father	0.01	-0.16–0.19	0.899
Woman, no child	0.09	-0.05–0.23	0.223
Man, no child	0.18	0.01–0.34	**0.039**
Observations	933		
R^2^ / R^2^ adjusted	0.005 / 0.002		

The only significant effect was higher overheating worry in men without children compared to mothers in the US. However, equivalence tests to estimate if there is really no effect of a certain variable, were inconclusive for the categories “Father”, “Woman, no child” in the UK (S2 Appendix).

#### Hypothesis 3

Hypothesis 3 tests if overheating worry is greater amongst parents of young children (0–2 years: N_UK_ = 178 [18.6%], N_US_ = 180 [18.9%]) than parents of older children (3–5 years, N_UK_ = 145 [15.2%], N_US_ = 120 [12.6%]; 6–10 years, N_UK_ = 86 [9.0%], N_US_ = 89 [9.4%]; 11–17 years, N_UK_ = 54 [5.7%], N_US_ = 60 [6.3%]) and non-parents (N_UK_ = 492 [51.5%]; N_US_ = 501 52.7%]) given that younger children are particularly affected by overheating. Table 2 shows the regression results for the UK sample (a) and the US sample (b).

**Table 2:** Relating overheating worry to age of youngest child. a) Regression results using UK sample (0–2 years reference category). b) Regression results using US sample (0–2 years reference category).

	**Overheating worry (UK)**		
*Predictors*	*Estimates*	*CI*	*p*
(Intercept)	2.02	1.90–2.14	<0.001
0–2 years	*Reference*		
3–5 years	-0.14	-0.32–0.04	0.124
6–10 years	-0.13	-0.33–0.08	0.235
11–17 years	0.12	-0.13–0.37	0.343
No child	-0.10	-0.24–0.03	0.142
Observations	955		
R^2^ / R^2^ adjusted	0.007 / 0.003		
	**Overheating worry (US)**		
*Predictors*	*Estimates*	*CI*	*p*
(Intercept)	1.94	1.81–2.07	**<0.001**
0–2 years	*Reference*		
3–5 years	0.16	-0.04–0.36	0.117
6–10 years	0.27	0.04–0.49	**0.020**
11–17 years	0.22	-0.03–0.48	0.090
No child	0.24	0.09–0.39	**0.001**
Observations	950		
R^2^ / R^2^ adjusted	0.012 / 0.008		

No predictor category was significant in the UK data; however, equivalence testing indicated that it could not be decided if there was truly a null effect of the various categories against the reference category (S3 Appendix). For US, overheating concern was significantly higher when the youngest child was 6–10 years or for non-parents. Equivalence testing rejected the null-hypothesis for 11–17 years and was undecided for 3–5 years (S3 Appendix).

Overall, hypothesis 3 was not supported by the data.

### Current and future actions against overheating

#### Hypothesis 4

Hypothesis 4a tests if parents are more likely to install / upgrade air-conditioning (AC) in the next three years, scored on a 7-point scale ranging from ‘very unlikely’ to ‘very likely’. Mean values for the UK in parents were M = 2.48 (SD = 1.85) and in non-parents M = 2.34 (SD = 1.75). The difference was not significant, *t*(923) = -1.22, *p* = .223. The equivalence test was significant (S3 Appendix). The equivalence test and the null-hypothesis test combined indicated that the observed effect was statistically not different from zero and statistically equivalent to zero. In the US, likelihood to install / upgrade AC was significantly higher in parents [M = 3.03 (SD = 2.15)] than non-parents (M = 2.64, 1.88)], t(856) = -2.78, *p* < .001.

Hypothesis 4b about uptake of electric fans was only tested in the UK given how prevalent they already are in the US. Mean uptake in parents was higher with M = 4.56 (SD = 2.00) than in non-parents, M = 4.21 (SD = 2.14). The difference was significant, *t*(949) = -2.64, *p* = .008, and Hypothesis 4b hence confirmed.

Hypothesis 4c about the uptake of external shading is only tested amongst those whose tenure is “owner” as those who rent a property are usually not allowed to add external shading to their home. For the UK, the difference was not significant, *t*(504) = -1.28, *p* = .202, M_parent_ = 2.57 (SD = 1.57), M_non-parent_ = 2.40 (SD = 1.47). The equivalence test was non-significant (S4 Appendix). Taken together, the equivalence test and the null-hypothesis test indicated that the observed effect is statistically not different from zero and statistically not equivalent to zero. For the US, the difference was not significant, *t*(429) = -1.44, *p* = .151, M_parent_ = 3.38 (SD = 1.91), M_non-parent_ = 3.11 (SD = 1.86). The equivalence test was non-significant (S4 Appendix). The equivalence test and the null-hypothesis test combined indicated that the observed effect is statistically not different from zero and statistically not equivalent to zero.

#### Hypothesis 5

Hypothesis 5 states that the number of mitigation options to fight overheating is higher in parents than non-parents on a normal summer day (Hypothesis 5a) and during a heatwave (Hypothesis 5b). In the UK, there were 204 (21.4%) respondents who did not experience any overheating during a normal summer and 87 (9.1%) who did not experience overheating during a heatwave. In the US, this was the case for 243 (25.6%) respondents during a normal summer and 190 (20.0%) during a heatwave. These were not asked about what coping actions they took.

**Fig 2:**
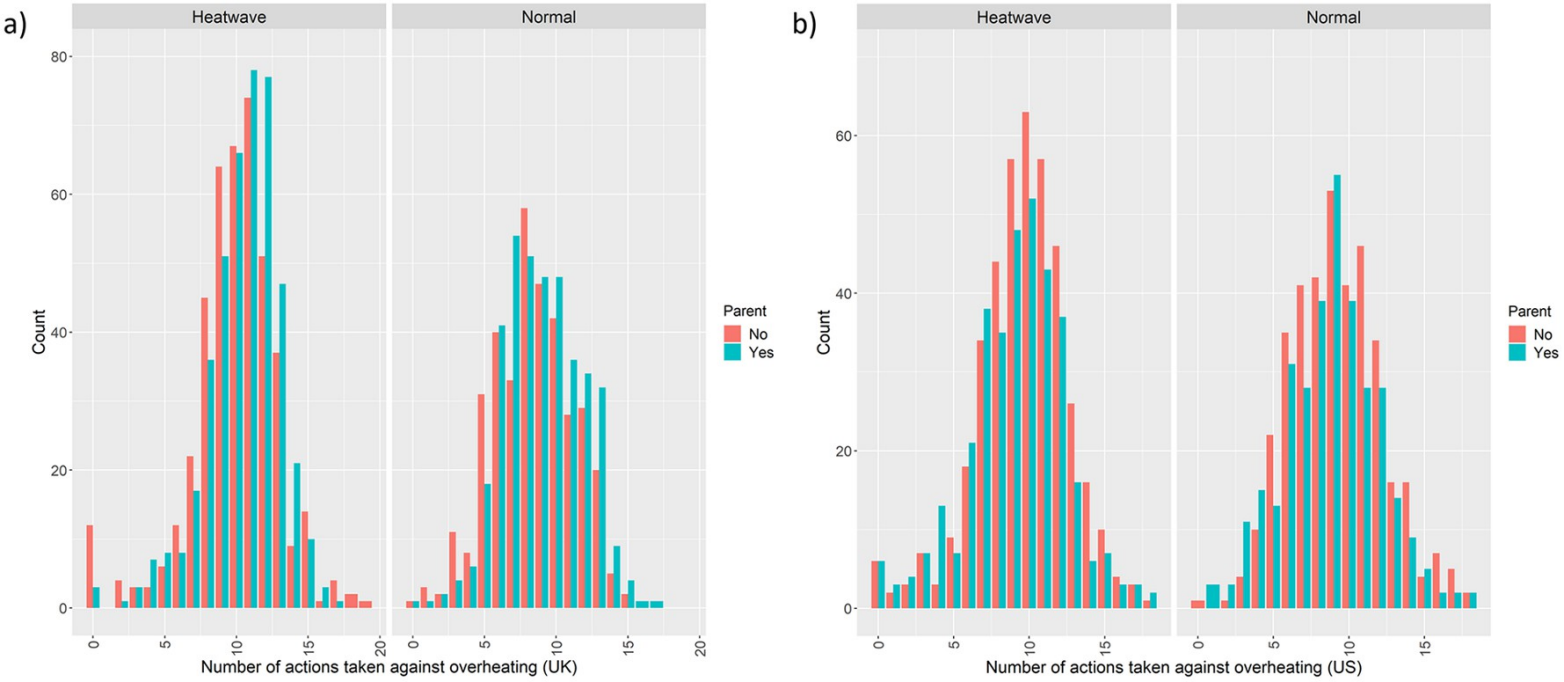
Frequency distribution of the number of overheating actions taken during a heatwave and a normal summer in the UK (a; top panel) and the US (b, bottom panel).

In the UK, parents took more actions against overheating than non-parents, both during normal summer (M_parent_ = 9.00, SD _parent_ = 2.75; M_non-parent_ = 8.4; SD _non-parent_ = 2.80) and during a heatwave (M_parent_ = 10.40, SD _parents_ = 2.58; M_non-parent_ = 9.91, SD _non-parent_ = 3.06), see also Fig 2. Parental status was a significant predictor for overheating actions in the UK, both in a normal summer and a heatwave (see Table 3 for the results of the Poisson regression).

**Table 3:** Predicting number of overheating actions in the UK.

	Normal summer (UK)			Heatwave (UK)		
*Predictors*	*Incidence Rate Ratios*	*CI*	*p*	*Incidence Rate Ratios*	*CI*	*p*
(Intercept)	8.39	8.10–8.69	**<0.001**	9.91	9.62–10.21	**<0.001**
No	*Reference*			*Reference*		
Yes	1.07	1.02–1.12	**0.006**	1.05	1.01–1.10	**0.019**
Observations	751			868		
R^2^ Nagelkerke	0.016			0.010		

In the US, parents carried out fewer actions against overheating than non-parents, both on a normal summer day (M_parent_ = 8.80, SD_parent_ = 3.11; M_non-parent_ = 9.27; SD _non-parent_ = 3.05) and during a heatwave (M_parent_ = 9.10, SD _parent_ = 3.23; M_non-parent_ = 9.66, SD _non-parent_ = 3.03), see Table 4 for the results of the Poisson regression.

**Table 4:** Predicting number of overheating actions in the US.

	Normal summer (US)			Heatwave (US)		
*Predictors*	*Incidence Rate Ratios*	*CI*	*p*	*Incidence Rate Ratios*	*CI*	*p*
(Intercept)	9.27	8.97–9.58	**<0.001**	9.66	9.36–9.96	**<0.001**
No	*Reference*			*Reference*		
Yes	0.95	0.90–1.00	**0.035**	0.94	0.90–0.99	**0.011**
Observations	707			760		
R^2^ Nagelkerke	0.009			0.012		

Hence, hypothesis 5 was confirmed in the UK but not the US.

### Exploratory analysis

The preregistered analyses painted a conflicting picture; only two of the hypotheses were confirmed, with other analyses inconclusive and others opposite to expectations.

#### Role of awareness of risks of overheating

One possible explanation is that there is a lack of awareness amongst parents around hot temperatures posing health risks to children. Whilst awareness of overheating risks was not assessed directly in this study, an open-ended question asked those who had any overheating worry why they were concerned about overheating, and to specify for whom (if not themselves).

In the UK, of the 392 parents who had at least some overheating worry, 116 (29.6%) gave their children’s health as a reason for being concerned about overheating; 276 (70.4%) did not mention children as to why they were concerned about overheating but gave other reasons. Mean overheating worry was higher in parents who mentioned their children’s health as a concern [M = 2.39 (SD = 0.78)] than in parents who did not mention it [M = 2.04 (SD = 0.75)]. This difference was significant, *t(*390) = 4.24, *p* = < .001.

In the US, of the 372 parents who were asked this question, i.e. those who had at least some overheating worry, 121 (32.5%) gave their children’s health as a reason for being concerned about overheating; 251 (67.5%) did not mention children as to why they were concerned about overheating but gave other reasons. Mean overheating worry was higher in parents who mentioned their children’s health as a concern [M = 2.47 (SD = 0.85)] than in parents who did not mention it [M = 2.20 (SD = 0.77)]. This difference was significant, *t*(370) = 2.98, *p* = .003.

Hence, in both countries, amongst parents with any overheating worry, those who mentioned their children’s health as reason for being concerned, had higher overheating worry.

#### Type of mitigation options

**Fig 3:**
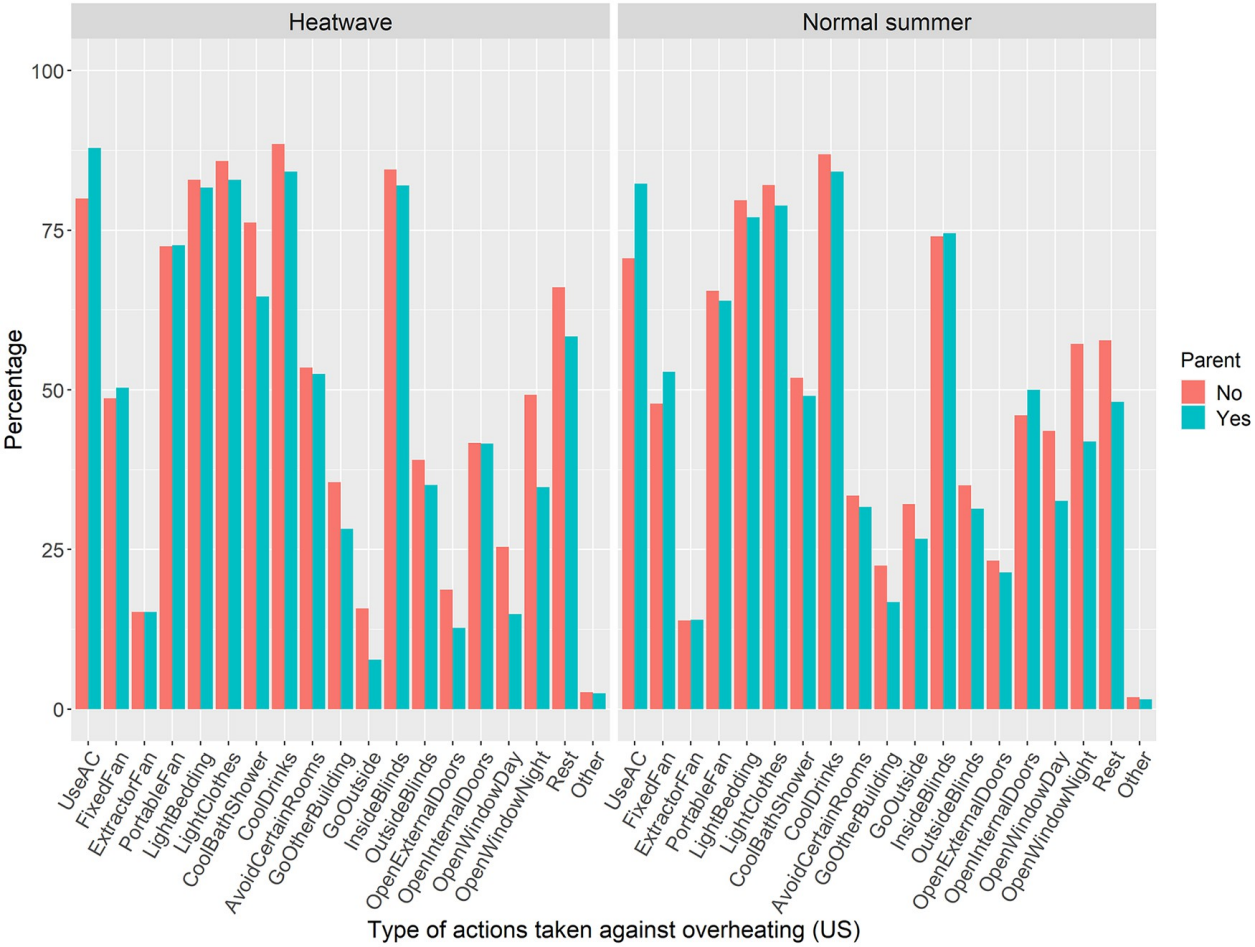
Type of overheating actions taken during a heatwave and a normal summer in the US, split up by parental status.

Testing hypothesis 5 had shown that in the UK, parents performed more actions against overheating than non-parents; however, this effect was not found in the US. One possible explanation is that in the US parents and non-parents used different mitigation options: Using an air-conditioning is very effective in cooling down a building, making it less necessary to perform other actions with lower impact. Hence, it was tested if parents in the US were more likely to use air-conditioning to deal with overheating than non-parents. Fig 3 shows all types of overheating options and their prevalence amongst parents and non-parents in the US sample.

During a normal summer, 70.6% of non-parents reported using air-conditioning compared to 82.3% of parents. In a heatwave, it was 80.0%% of non-parents and 87.9% of parents. A chi-square test of independence was performed to examine the relation between parental status and air-conditioning use, separately for a normal summer’s day and a heatwave. The relation between these variables was significant in both cases; normal summer: *X*^*2*^ (1, *N* = 696) = 12.38, *p* < .001; heatwave: *X*^*2*^ (1, *N* = 696) = 7.40, *p* = .006. In the US, parents were more likely to use air-conditioning to deal with overheating than non-parents.

The analysis was not repeated for the UK given the very low prevalence and usage of AC.

**Fig 4:**
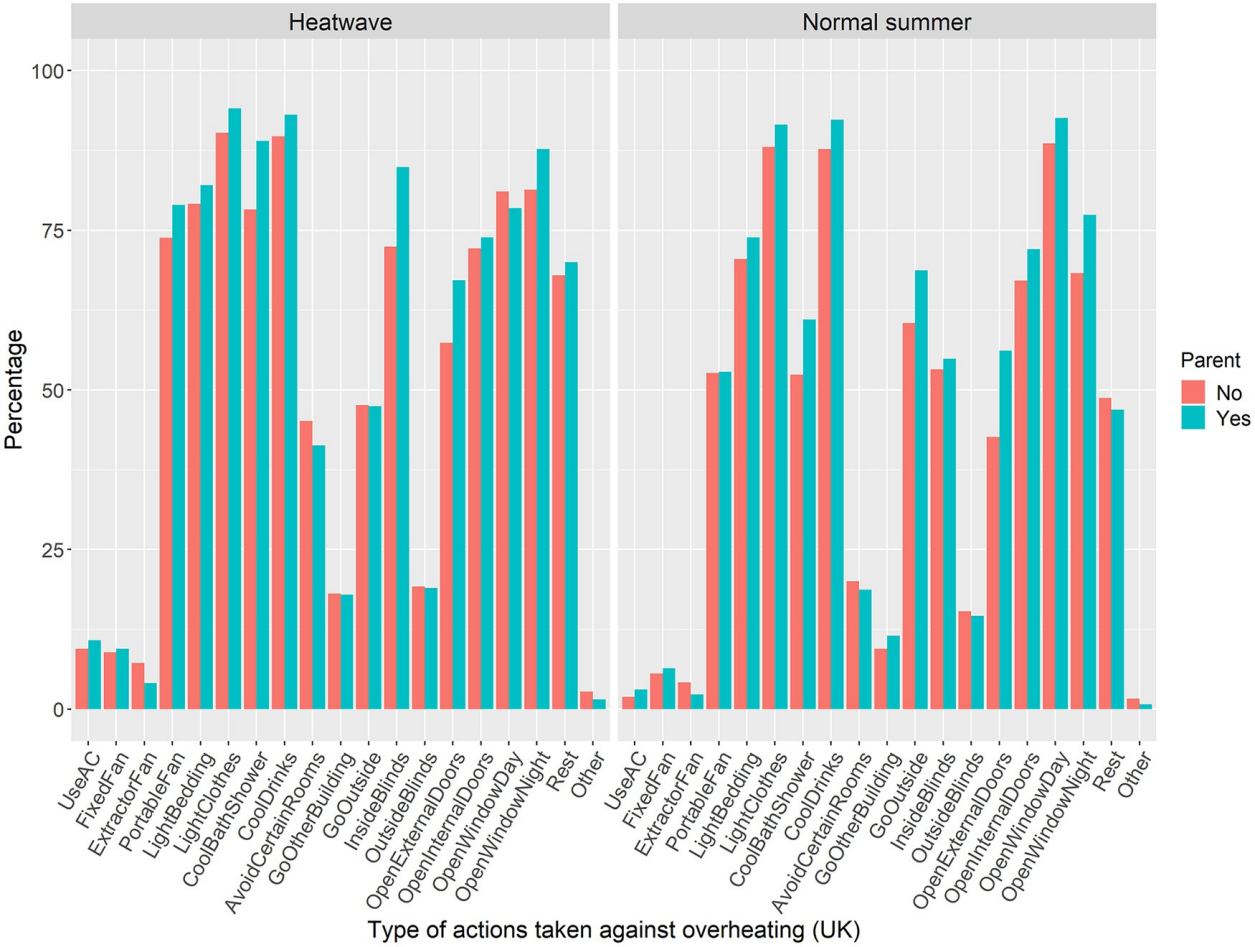
Type of overheating actions taken during a heatwave and a normal summer in the UK, split up by parental status.

Fig 4 shows the overheating actions performed in the UK for comparison purposes.

#### Controlling for other confounders

Parents and non-parents differed in some characteristics that might be related to overheating worry. Hence, an explanatory analysis was run to test if other factors impacted on worry about overheating, including dwelling type, tenure, age of respondent, experience of overheating, employment status and general health (Table 5).

**Table 5:** Relating overheating worry to other variables. a) Regression results UK sample. b) Regression results US sample.

*Predictors*	**Overheating worry (UK)**		
	*Estimates*	*CI*	*p*
(Intercept)	1.53	1.32–1.73	**<0.001**
*Parent No*	*Reference*		
Parent Yes	-0.01	-0.12–0.10	0.845
*Dwelling Type*: *Semi-detached*	*Reference*		
Terraced house	-0.18	-0.29 –-0.06	**0.004**
Detached house / bungalow	0.03	-0.10–0.16	0.630
Purpose-built flat	-0.02	-0.17–0.13	0.821
Converted flat	-0.01	-0.23–0.21	0.951
*Tenure*: *Own with mortgage*	*Reference*		
Rent it privately	0.12	0.00–0.23	**0.043**
Social housing	0.25	0.08–0.42	**0.005**
Own outright	0.06	-0.13–0.24	0.561
Other	0.17	-0.07–0.42	0.160
*Overheating in summer*: *No*	*Reference*		
In one room	0.42	0.24–0.59	**<0.001**
In multiple rooms	0.73	0.62–0.85	**<0.001**
In all rooms	1.18	1.03–1.33	**<0.001**
*Age respondent*: *25–29 years*			
30–34 years	-0.12	-0.23 –-0.00	0.049
35–39 years	-0.07	-0.20–0.06	0.280
40–44 years	-0.09	-0.24–0.06	0.224
*Employment*: *> = 30 hours/week*	*Reference*		
Less than 30 hours/week	0.06	-0.06–0.18	0.347
Other	0	-0.20–0.20	0.993
Unemployed	-0.11	-0.31–0.10	0.300
Student	-0.08	-0.32–0.16	0.512
Parental leave	0.2	-0.06–0.46	0.131
Not working; disability/ illness	0.22	-0.08–0.52	0.145
*General health*	-0.06	-0.10 –-0.02	**0.005**
Observations	941		
R^2^ / R^2^ adjusted	0.269 / 0.251		
*Predictors*	**Overheating worry (US)**		
	*Estimates*	*CI*	*p*
(Intercept)	1.49	1.27–1.71	**<0.001**
*Parent*: No	*Reference*		
Parent Yes	-0.02	-0.13–0.09	0.73
*Dwelling Type*: Detached	*Reference*		
Apartment / flat	0.07	-0.06–0.21	0.302
Semi-detached house	0.06	-0.16–0.27	0.612
Terraced house	0.19	-0.05–0.43	0.119
Other	0.25	-0.04–0.54	0.088
Manufactured/mobile home	0.09	-0.20–0.39	0.531
*Tenure*: Rent it	*Reference*		
Own with a mortgage	-0.05	-0.19–0.08	0.416
Own outright	0.28	0.09–0.46	**0.004**
Other	-0.04	-0.26–0.18	0.702
*Overheating in summer*: No	*Reference*		
In one room	0.78	0.63–0.93	**<0.001**
In multiple rooms	0.91	0.78–1.03	**<0.001**
In all rooms	1.26	1.12–1.40	**<0.001**
*Age respondent*: 25–29 years	*Reference*		
30–34 years	0.09	-0.03–0.21	0.161
35–39 years	0	-0.14–0.14	0.987
40–44 years	0	-0.18–0.17	0.956
*Employment*: > = 30 hours/week	*Reference*		
Less than 30 hours/week	0.03	-0.12–0.17	0.738
Unemployed	0.05	-0.10–0.20	0.527
Not working other reason	-0.05	-0.21–0.12	0.594
Student	-0.06	-0.26–0.13	0.52
Other	0.07	-0.25–0.39	0.678
*General health*	-0.05	-0.10 –-0.01	**0.024**
Observations	947		
R^2^ / R^2^ adjusted	0.315 / 0.300		

Experiencing overheating was significantly associated with greater overheating worry. Being in better health was associated with a decrease in overheating worry. Owning outright was associated with greater overheating worry than renting in the US, whereas those living in rented accommodation in the UK had lower overheating worry. However, controlling for other covariates that differed between parents and non-parents did not change the finding that parents were not more concerned about overheating than non-parents. However, the analysis emphasized the importance of experiencing overheating for being worried about overheating. The number of respondents who reported overheating was high as shown in Table 6.

**Table 6:** Percentage of respondents reporting experiencing overheating during heatwaves and a normal summer in the US and the UK.

	US		UK	
	Heatwave	Summer	Heatwave	Summer
No Overheating	20.0%	25.6%	9.1%	21.4%
In one room	14.2%	16.1%	9.1%	10.2%
In multiple rooms	33.3%	35.1%	51.8%	51.9%
In all rooms	32.5%	23.36%	30.0%	16.5%

## Discussion

Two online survey studies tested to what extent parents are more worried about overheating than non-parents in the UK and the US. The first three hypotheses were not confirmed in either country: Parents, mothers, and those of young children were not more worried about overheating than other respondents. Two hypotheses relating to the uptake of mitigation options were confirmed: Parents in the US were more likely to say they would upgrade / install additional AC in the next three years and parents in the UK said they would be more likely to install electric fans in the future. Hypothesis 5 on number of mitigation options against overheating was confirmed in the UK: parents in the UK use more mitigation options than non-parents both during heatwaves and on a regular summer’s day. This effect was not present in the US. However, analysis of the US data showed that parents were more likely to use AC to deal with overheating than-non parents which is the most effective of all options listed and might explain why parents use fewer options overall. As also reported by Nicholls and Strengers [33], a wide range of mitigation options against overheating were used, with about 9–10 actions mentioned on average.

Exploratory analysis also showed that those parents who mentioned children’s health as a reason for overheating worry worried more about overheating than parents who gave other reasons for being concerned about overheating. High temperatures are an objective health risks for children, especially infants. However, not all parents mentioned it which could indicate a lack of awareness of these risks and hence a lesser worry about overheating. This means future research needs to assess actual awareness of overheating health risks and assess if this mediates overheating worry.

Further exploratory work showed that also controlling for other covariates, such as employment status, age, dwelling type and tenure did not change the effect of being a parent vs. not. Being in better health was associated with lower overheating worry. This might reflect a realistic awareness of risks of overheating given that those in poorer health are more vulnerable to overheating [37,38]. However, it might also reflect a generally higher level of concern, not specific to overheating; the current analysis did not test this.

Analysis also showed that experiencing overheating is a strong predictor for worry about overheating. Overheating is already on the rise in places with temperate climates that still have low prevalence of air-conditioning [15,39]. Already in this survey, 80–90% of respondents reported experiencing overheating during heatwaves. Given the predicted increase in temperatures and in frequency, severity and duration of heatwaves [40] it is likely that more and more overheating in homes will be experienced. Hence, overheating worry might grow accordingly, causing repeated stress to occupants and potentially leading to an increase in mental health problems [41]. Increase in experienced overheating may also lead to an increase in active cooling. Whilst estimates on uptake on future cooling vary widely, studies agree it will become more common [42,43]. This paper adds the novel insight that parents indicate a higher likelihood of adding or upgrading active cooling. Hence, having children could become a trigger point for an increase in electricity use for cooling purposes.

The proportion of self-reported overheating by respondents in these studies was higher than previous estimates using temperature-based estimates [16,44]; indicating a potential mismatch between calculated and perceived overheating.

Limitations include the studies not being nationally representative and relying on stated perception and stated preference. Also, younger participants were more prevalent in the non-parent group than the parent group and the groups might differ in other important characteristics that were not assessed. For example, income was not measured but might have been different between groups. Another variable that might have been particularly important within the group of parents was awareness of the health consequences of overheating for children. Further research needs to test if awareness of health risks for children is a necessary precondition for greater overheating worry in more representative samples. Future work could also explore to what extent the exposure to overheating, such as through different amount of time spent at home, impacts on overheating worry.

In summary, the main hypotheses around greater overheating worry in parents were not supported by this study. However, parental status might have implications for cooling energy use.

## Methods

### Ethics information

The studies have been approved by the UCL BSEER ethics committee that assesses low-risk ethics applications (approval number: 20210625_IEDE_STA_ETH). Written informed consent was obtained from all participants at the beginning of the survey (see S5 Appendix); the survey could only be started after participants indicated their consent by ticking the relevant boxes. The expected duration of the survey was 12 minutes. Participants were paid pro-rata of an hourly rate of £7.50/hr ($9.60/hr), the recommended rate of Prolific, i.e. for 12 minutes, this is £1.50.

### Design

Two online survey studies were run for this paper with participants from the UK and the US who are signed up to the platform Prolific. Prolific is an online platform that enables anonymous data collection by connecting researchers to participants. Anyone over 18 who lives in a certain country, i.e. most OECD countries, can sign up to Prolific for free. Once signed up, they will be able to see online studies and choose to participate in them. Payment is handled by Prolific, i.e. the researcher transfers money to Prolific who then distributes payment to participants. Prolific provides high quality data [45], was comparable to a national representative survey for questions around user perceptions and experiences [46] and showed similar retention performance to traditional cohort studies [47]. The studies in this paper were observational in nature; i.e. participants are not randomly assigned to conditions and no blinding was used.

#### Participants and sample size

**Fig 5:**
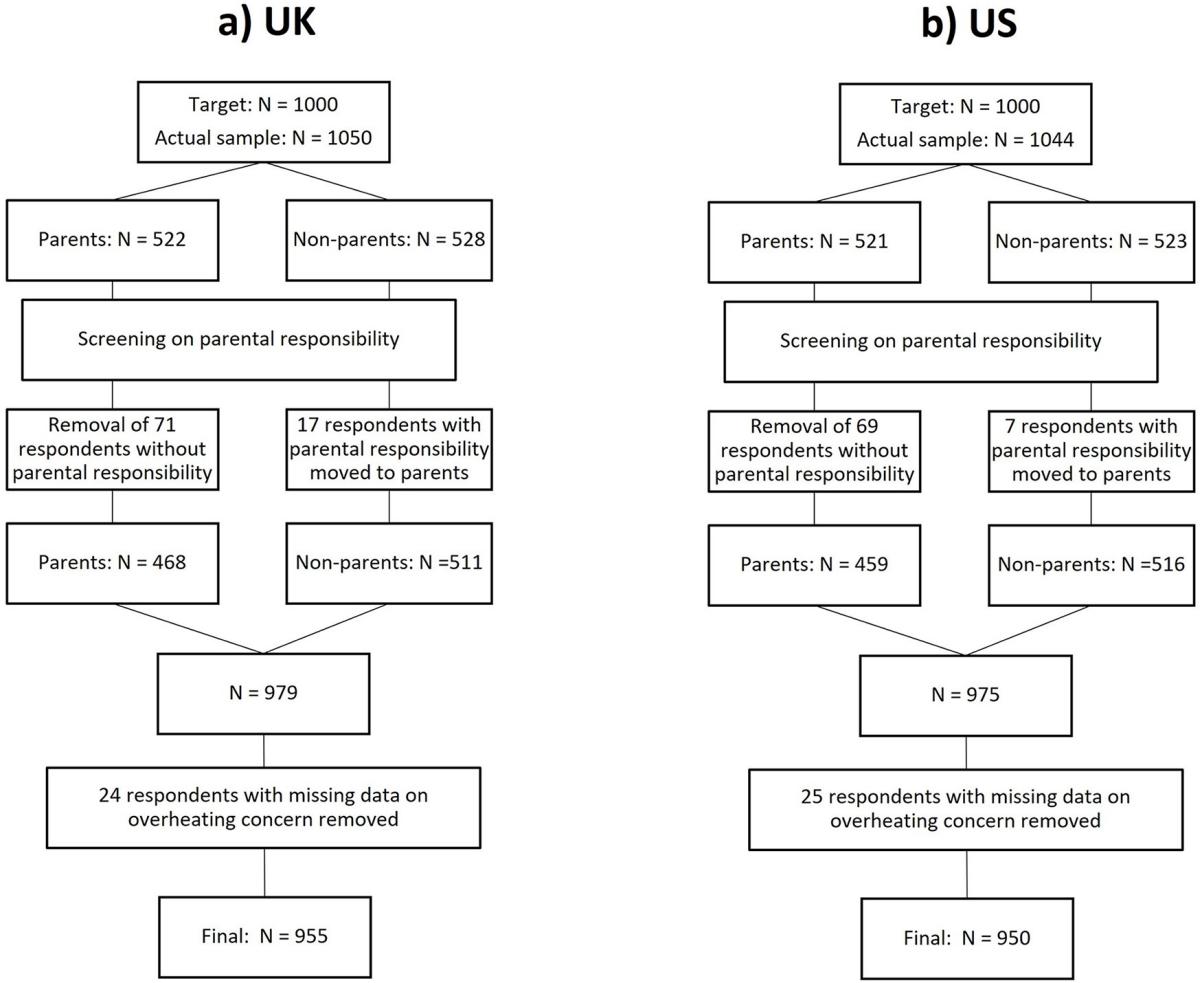
Diagram of data exclusion for the UK (a) and the US (b) to arrive at the final sample size.

The target sample size was N = 1000 in each study. Prolific as background data collects information on whether participants have children or not. 500 participants in each study were supposed to have children and 500 not to. The age range was restricted to 25–44 years, i.e. the age in which most people have children. UK participants could reside in any part of the country. In the US, participants had to be based in one of the following US states: Washington, Oregon, California, Arizona, Nevada, Idaho, Montana, Wyoming, Utah, Colorado, New Mexico, Texas, Oklahoma, Kansas, Nebraska, South Dakota, North Dakota; some of which experienced an extraordinary heatwave in 2021 that has been made more likely because of climate change [48]. Fig 5 shows development of the final sample size.

#### Sampling plan

For all analyses, a significance level alpha = 0.05, power beta = 0.95 have been chosen. A small effect size was assumed for all analyses. The maximum required sample size for any of the analyses is N = 934; however, sample size was set to N = 1000 in each study as some missing data was expected. Sample size calculation was carried out in R [49] using the packages *WebPower* [50] and *pwr* [51]. Table 7 shows details for all planned hypotheses testing. All testing was carried out two-sided. If any of the hypothesis was not confirmed, frequentist equivalence testing was used to understand if a non-significant effect suggested the absence of an effect or an inconclusive finding [52,53] using the R packages *TOSTER* [53], *parameters* [54], and *bayestestR* [55].

**Table 7:** Overview of hypotheses, power calculations and analysis plan.

Hypothesis	Sampling plan (power analysis)	Analysis Plan
H1: Parents are more worried about overheating than non-parents.	α = 0.05; β = 0.95; d = 0.25; necessary N = 834	Independent sample t test; Exclusion criteria: choosing ‘prefer not to say’ for any of the overheating worry items
H2: Mothers are more worried about overheating than fathers or non-parents.	α = 0.05; β = 0.95; f = 0.02; necessary N = 934	OLS regression; parental status as predictor: mother (reference category), father, female non-parent; male non-parent
H3: Parents of younger children are more worried about overheating than non-parents or parents with older children.	α = 0.05; β = 0.95; f = 0.02, necessary N = 934	OLS regression; age of youngest child used to indicate age category; 0–2 years (reference); 3–5 years; 6–10 years; 11–17 years; no children
H4a(UK): Parents indicate higher likelihood to install air-conditioning in the future than non-parents. H4a(US): Parents indicate higher likelihood to install a new or upgrade an existing air-conditioning system than non-parents.	α = 0.05; β = 0.95; f = 0.02, necessary N = 934	Independent sample t test; Exclusion criteria (UK only): already have an air-conditioning system.
H4b (UK only): The likelihood to install electric fans is higher in parents than non-parents (UK only) mitigation options	α = 0.05; β = 0.95; f = 0.02, necessary N = 934	Independent sample t test
H4c: The likelihood to install external shading is higher in parents than non-parents.	α = 0.05; β = 0.95; f = 0.02, necessary N = 934	Independent sample t test; Exclusion criteria: Renters
Hypothesis 5a: Parents use more mitigation options against overheating than non-parents during a normal summer. H5b: Parents use more mitigation options against overheating than non-parents during a heatwave.	exp0 = 1.2343, exp1 = 1.5236; α = 0.05; β = 0.95; necessary N = 579	Poisson regression; parents versus non-parents Exclusion criteria: Never experiencing overheating during a normal summer (for H5a) or during a heatwave (for H5b).

*Hypotheses*. The first research questions was addressed through three hypotheses where the outcome variable was always self-reported worry about overheating. Hypothesis 1 states that parents have greater overheating worry than non-parents. Hypothesis 2 postulates that mothers are more worried about overheating than fathers or those without children. Hypothesis 3 splits parenting status up depending on the age of children and states that overheating worried is greater amongst parents of young children (0–2 years) than parents of older children and non-parents.

The second research question tests if parents are more likely to install cooling devices and use more mitigation options than non-parents. The analysis varied slightly between the US and the UK sample given the already very high prevalence of air-conditioning in the US. Hypothesis 4a states that the likelihood to install air-conditioning is higher in parents than non-parents (UK) or that the likelihood to upgrade an existing air-conditioning system or to get an initial one is higher in parents than non-parents (US). Hypothesis 4b states that likelihood to install electric fans is higher in parents than non-parents (UK only) and hypothesis 4c states that that the likelihood to install external shading is higher in parents than non-parents.

Those who do experience overheating were asked which mitigation options if any they employed. Hypothesis 5 states that the number of mitigation options to mitigate overheating is higher in parents than non-parents on a normal summer day (Hypothesis 5a) and during a heatwave (Hypothesis 5b).

### Coding of variables

#### Development of outcome variables

The outcome variable used to test the first three hypotheses is the mean worry about overheating. Since no scale exists to measure worry about overheating, I adapted an existing scale that was originally used to measure worry about cancer [56] and then to measure worry about Covid-19 [57]. The items were changed to ask about overheating (Table 8). The answer options were 1 (*never / rarely*), 2, 3, 5 (*almost always / continuously*) and *prefer not to say*.

**Table 8:** Items to assess overheating worry.

Items to assess overheating worry
During this summer, how often have you thought about your home overheating?
During this summer, has thinking about your home overheating affected your mood?
During this summer, has thinking about the possibility of your home overheating affected your capacity to perform your “everyday activities”?
How often do you worry about the possibility of your home overheating?
Is being worried about your home overheating an important problem for you?
To what degree does the possibility of your home overheating worry you?

Anyone who chose *prefer not to say* for any of the six items was excluded. Cronbach’s was α = 0.92 (95% CI: 0.91–0.92) in the UK and α = 0.93 (95% CI: 0.92–0.94) in the US; hence, the scores for the six items were averaged for each participants.

To test hypotheses 4 a, b, c, the outcome variable is the ‘likelihood to install cooling devices in the home‘ judged on a scale from 1 (very unlikely) to 7 (very likely). The exact phrasing varies slightly across countries (Table 9).

**Table 9:** Items to assess likeliness to install cooling devices.

Hypothesis	Country	Outcome variable	Exclusion criteria
4a	UK	In the next three years, how likely are you to get an air conditioning system (portable or fixed) for your home?	*Already have one*
4a	US	In the next three years, how likely are you to get an air conditioning system (portable or fixed) for your home? OR in the next three years, how likely are you to upgrade the air conditioning system you have in your home?	None
4b	UK & US	In the next three years, how likely are you to get external shading for your home, e.g. external shutters, an awning or a tree?	Renters
4c	UK	In the next three years, how likely are you to get an electric fan / additional electric fans for your home?	None

Because of an coding error in the survey, the response points of 1 and 2 were swapped for the question on external shading, i.e. the scale was shown as *2 (very unlikely)*, *1*, *3*, *4*, *5*, *6*, *7 (very likely)*. Since it cannot be estimated if participants should 2 or 1 to indicate very unlikely, both answers were recoded as 1.5.

The outcome variable for Hypotheses 5a and 5b is the ‘number of mitigation actions’ employed. The question asks “Please say which of these things, if any, you or your household sometimes do to when your home overheats during a typical summer and during a heatwave?” and participants could tick all of the 19 answer options that apply (see S5 Appendix). The number of responses were summed up for a typical summer (Hypothesis 5a) and a heatwave (Hypothesis 5b). If someone chose the option of “other”, this was counted as one entry even if the person specified more than one option in their open-ended response.

#### Developed predictor variables

One predictor variable to be developed was the coding of each respondent as having parental responsibility or not. Every respondent was asked if they have parental responsibility. If anyone from the group pre-selected not to have children ticked parental responsibility, they were considered as a parent and it was assumed that they omitted this detail in their initial registration / have not updated it. If anyone pre-selected to have children did not tick parental responsibility for any child, they are excluded from further analysis.

The same survey question was also be used to develop a variable standing for the age of the youngest child. The question asks in which age category the respondent has children (0 years, 1–2 years, 3–5 years, 6–10 years, 11–17 years). The choice indicating the youngest child was used to develop the variable “age of youngest child” (0–2 years, 3–5 years, 6–10 years, 11–17 years, not applicable).

The third predictor variable combined information about the gender of the respondent and their parental status. Those who indicate to be female and have children were coded as ‘mother’, those male and have children as ‘father’, those female and without children as “female, no children’, those male and without children as ‘male, no children’. Since there were fewer than 25 participants who were coded as ‘other’ or ‘prefer not to say’ they were excluded for the analysis of Hypothesis 3 but retained for all other analyses that did not include gender.

*Statistical tests*. Analysis and visualization were carried out in R using base statistics functions and *ggplot* [58], using use a t-test (Hypothesis 1, Hypothesis 3), ordinary least square regression (Hypothesis 2, 4) and Poisson regression (Hypothesis 5). If any of the hypothesis was not confirmed, frequentist equivalence testing was used to understand if a non-significant effect suggests the absence of an effect or an inconclusive finding [52,53] using the R packages *TOSTER* [53], *parameters* [54], and *bayestestR* [55].

The analysis was prespecified prior to data collection to avoid confirmatory bias. Exploratory analysis is explicitly labelled as such in this paper.

## Supporting information

S1 Table Further demographic information about the participants: Employment, tenure, dwelling type. (DOCX)

S1 Appendix Equivalence testing for hypothesis 1. (DOCX)

S2 Appendix Equivalence testing for hypothesis 2. (DOCX)

S3 Appendix Equivalence testing for hypothesis 3. (DOCX)

S4 Appendix Equivalence testing for hypothesis 4. (DOCX)

S5 Appendix Copies of the surveys used. (PDF)

S6 Appendix STROBE reporting guideline. (DOCX)

S7 Appendix TReQ statement to show details on pre-registration, data, code, and reporting guideline. (DOCX)
