# Supplementary material for: The role of parenthood in worry about overheating in homes in the UK and the US and implications for energy use: An online survey study
Source: PLoS One. 2022 Dec 1;17(12):e0277286. doi: 10.1371/journal.pone.0277286 (PMC9714918; doi:10.1371/journal.pone.0277286)
Supplement: S1 Table — (DOCX) [file pone.0277286.s001.docx]

S1 Table 1. Employment status (a), tenure (b), and dwelling type (c) in the sample split up by country and parental status, in percentages.

1. Employment status.

|  | US | | UK | |
| --- | --- | --- | --- | --- |
|  | Non-parent | Parent | Non-parent | Parent |
| Not working: longstanding disability or illness | 2 | 1.56 | 4.47 | 1.08 |
| Not working for other reasons | 2.59 | 12.03 | 0.81 | 7.78 |
| Other/prefer not to say | 2 | 0.22 | 1.82 | 2.38 |
| Student | 8.98 | 3.79 | 6.5 | 1.3 |
| Unemployed | 14.97 | 8.69 | 5.89 | 4.75 |
| Working (paid or unpaid): 30 hours a week or more | 56.89 | 57.02 | 70.33 | 46.22 |
| Working (paid or unpaid): less than 30 hours a week | 12.57 | 12.47 | 10.16 | 29.81 |
| Parental / adoption leave | | 1.78 |  | 6.7 |

b. Tenure. For the US, renting was not differentiated between social housing and privately rented, and is here presented all as “rent it privately”.

|  | US | | UK | |
| --- | --- | --- | --- | --- |
|  | Non-parent | Parent | Non-parent | Parent |
| Social housing | n/a | n/a | 7.72 | 11.66 |
| Own with a mortgage | 25.35 | 48.78 | 36.79 | 56.16 |
| Rent it privately | 57.49 | 38.53 | 37.2 | 28.51 |
| Own outright | 8.58 | 9.58 | 10.98 | 2.81 |
| Live here rent-free | 8.18 | 2.67 | 5.28 | 0.43 |
| Other | 0.4 | 0.45 | 2.03 | 0.43 |

c. Dwelling type. For the UK, mobile home was not assessed and ‘apartment / flat’ has the combined percentage for purpose built and converted flat.

|  | US | | UK | |
| --- | --- | --- | --- | --- |
|  | Non-parent | Parent | Non-parent | Parent |
| A detached house / bungalow | 44.71 | 62.36 | 15.45 | 21.17 |
| A manufactured/mobile home | 2 | 3.34 | n/a | n/a |
| A semi-detached house | 5.19 | 5.79 | 27.64 | 38.44 |
| A terraced house (including end terrace) | 3.79 | 4.45 | 22.56 | 28.51 |
| An apartment, flat | 41.32 | 21.38 | 32.52 | 11.23 |
| Other | 2.99 | 2.67 | 1.83 | 0.65 |
