# Supplementary material for: The role of parenthood in worry about overheating in homes in the UK and the US and implications for energy use: An online survey study
Source: PLoS One. 2022 Dec 1;17(12):e0277286. doi: 10.1371/journal.pone.0277286 (PMC9714918; doi:10.1371/journal.pone.0277286)
Supplement: S1 Appendix — (DOCX) [file pone.0277286.s002.docx]

S2 Appendix. Equivalence testing for Hypothesis 1.

**UK data**

**Hypothesis 1**

The equivalence test was significant, t(953) = 2.891, p = 0.00196, given equivalence bounds of -0.202 and 0.202 (on a raw scale) and an alpha of 0.05.


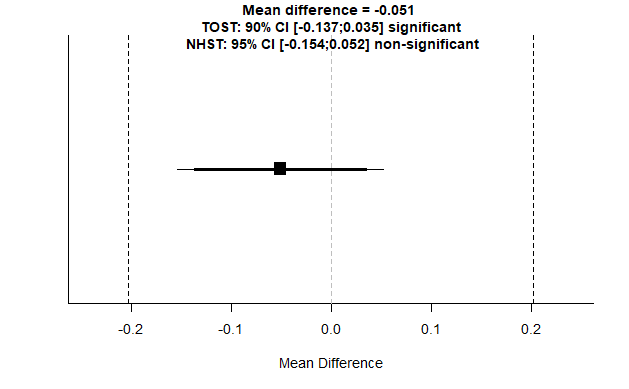


Figure S2. Observed mean difference in overheating concern between parents and non-parents (in raw scale units), the equivalence bounds (also in raw scores), and the 90% and 95% CIs.
