# Supplementary material for: The role of parenthood in worry about overheating in homes in the UK and the US and implications for energy use: An online survey study
Source: PLoS One. 2022 Dec 1;17(12):e0277286. doi: 10.1371/journal.pone.0277286 (PMC9714918; doi:10.1371/journal.pone.0277286)
Supplement: S2 Appendix — (DOCX) [file pone.0277286.s003.docx]

**S3 Appendix. Equivalence testing for Hypothesis 2.**

**UK data.**

Table S3a. Equivalence testing. Note, region of practical equivalence (ROPE): [-0.08 0.08].

| Parameter | 90% CI | % in ROPE | H0 | *p* |
| --- | --- | --- | --- | --- |
| (Intercept) | [ 1.90, 2.03] | 0% | Rejected | > .999 |
| ParentGender [Father] | [-0.17, 0.17] | 48.41% | Undecided | 0.426 |
| ParentGender [Woman, no child] | [-0.12, 0.08] | 80.65% | Undecided | 0.203 |
| ParentGender [Man, no child] | [-0.26, 0.00] | 30.82% | Rejected | 0.740 |


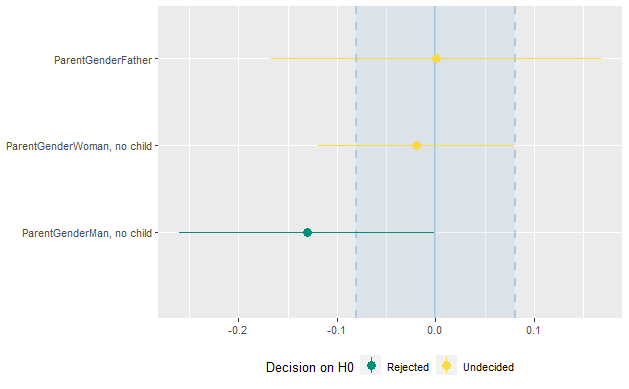


Figure S3a. Graphical representation of relationship of coefficient estimates to ROPE region and decision on H0.

**US data**

Table S3b. Equivalence testing. Note, region of practical equivalence (ROPE): [-0.09 0.09].

| Parameter | 90% CI | % in ROPE | H0 | *p* |
| --- | --- | --- | --- | --- |
| (Intercept) | [ 1.98, 2.15] | 0% | Rejected | > .999 |
| ParentGender [Father] | [-0.13, 0.16] | 60.36% | Undecided | 0.324 |
| ParentGender [Woman, no child] | [-0.03, 0.20] | 50.69% | Undecided | 0.498 |
| ParentGender [Man, no child] | [ 0.04, 0.31] | 18.70% | Rejected | 0.850 |


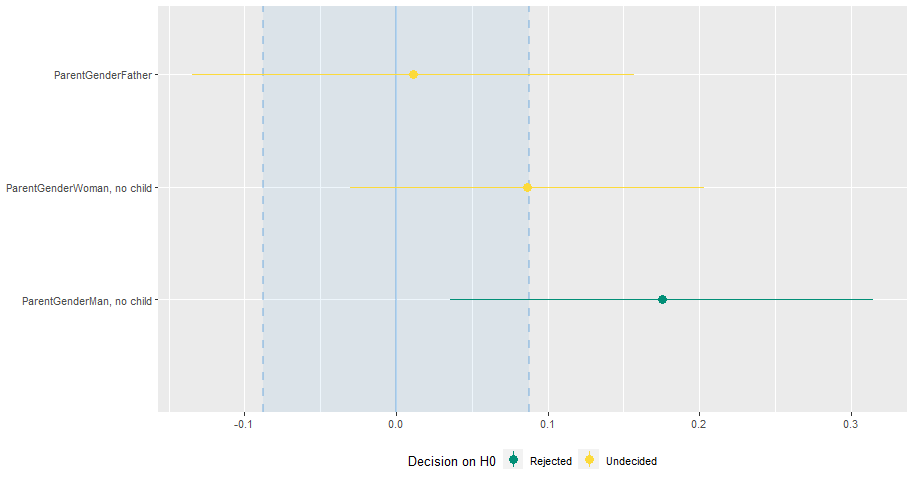


Figure S3b. Graphical representation of relationship of coefficient estimates to ROPE region and decision on H0.
