# Supplementary material for: The role of parenthood in worry about overheating in homes in the UK and the US and implications for energy use: An online survey study
Source: PLoS One. 2022 Dec 1;17(12):e0277286. doi: 10.1371/journal.pone.0277286 (PMC9714918; doi:10.1371/journal.pone.0277286)
Supplement: S3 Appendix — (DOCX) [file pone.0277286.s004.docx]

**S4 Appendix. Equivalence testing for Hypothesis 3.**

**UK**

Table S4a. Equivalence testing. Note, region of practical equivalence (ROPE): [-0.08 0.08].

| Parameter | 90% CI | % in ROPE | H0 | *p* |
| --- | --- | --- | --- | --- |
| (Intercept) | [ 1.92, 2.12] | 0% | Rejected | > .999 |
| AgeYoungest [3 - 5 years] | [-0.29, 0.01] | 30.47% | Undecided | 0.747 |
| AgeYoungest [6 - 10 years] | [-0.30, 0.05] | 37.06% | Undecided | 0.691 |
| AgeYoungest [11 - 17 years] | [-0.09, 0.33] | 39.15% | Undecided | 0.675 |
| AgeYoungest [No child] | [-0.22, 0.01] | 40.12% | Undecided | 0.632 |


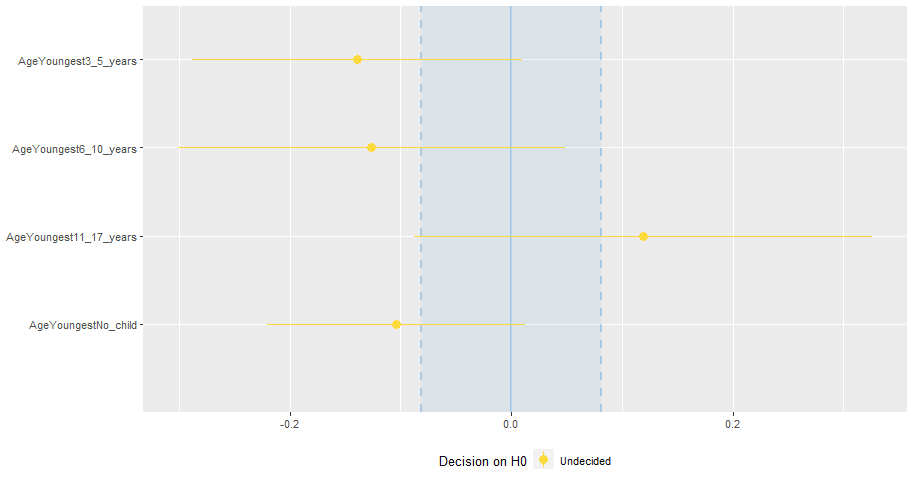


Figure S4a. Graphical representation of relationship of coefficient estimates to ROPE region and decision on H0.

**US**

Table S4b. Equivalence testing. Note, region of practical equivalence (ROPE): [-0.09 0.09].

| Parameter | 90% CI | % in ROPE | H0 | *p* |
| --- | --- | --- | --- | --- |
| (Intercept) | [ 1.84, 2.05] | 0% | Rejected | > .999 |
| AgeYoungest [3 - 5 years] | [-0.01, 0.33] | 28.28% | Undecided | 0.771 |
| AgeYoungest [6 - 10 years] | [ 0.08, 0.45] | 2.47% | Rejected | 0.942 |
| AgeYoungest [11 - 17 years] | [ 0.01, 0.44] | 18.93% | Rejected | 0.856 |
| AgeYoungest [No child] | [ 0.12, 0.37] | 0% | Rejected | 0.979 |


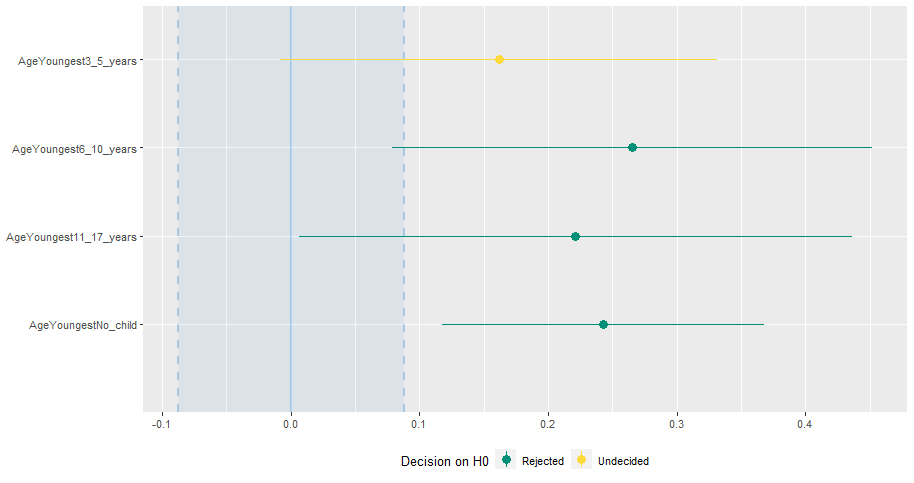


Figure S4b. Graphical representation of relationship of coefficient estimates to ROPE region and decision on H0.
