# Supplementary material for: The role of parenthood in worry about overheating in homes in the UK and the US and implications for energy use: An online survey study
Source: PLoS One. 2022 Dec 1;17(12):e0277286. doi: 10.1371/journal.pone.0277286 (PMC9714918; doi:10.1371/journal.pone.0277286)
Supplement: S4 Appendix — (DOCX) [file pone.0277286.s005.docx]

**S5 Appendix. Equivalence testing for hypothesis 4.**

UK

**Hypothesis 4 a**

The equivalence test was significant, t(953) = 2.622, p = 0.00444, given equivalence bounds of -0.451 and 0.451 (on a raw scale) and an alpha of 0.05.


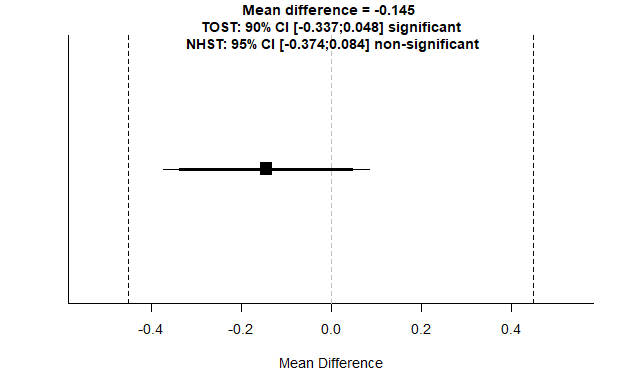


Figure S5a. Observed mean difference in likelihood to adapt air-conditioning between parents and non-parents (in raw scale units), the equivalence bounds (also in raw scores), and the 90% and 95% CIs.

**Hypothesis 4c**

The equivalence test was non-significant, t(506) = 1.530, p = 0.0633, given equivalence bounds of -0.382 and 0.382 (on a raw scale) and an alpha of 0.05.


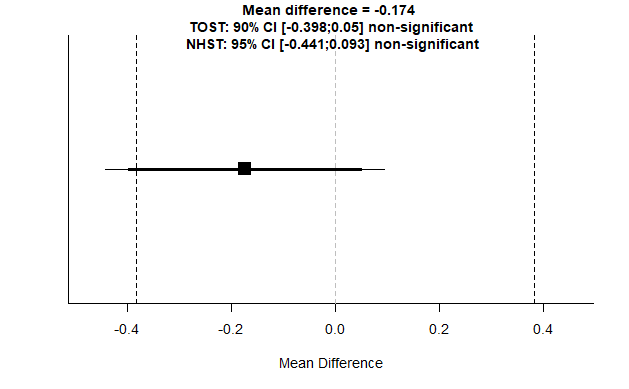


Figure S5b. Observed mean difference in likelihood to get external shading between parents and non-parents (in raw scale units), the equivalence bounds (also in raw scores), and the 90% and 95% CIs.

**US**

**Hypothesis 4c**

The equivalence test was non-significant, t(430) = 1.099, p = 0.136, given equivalence bounds of -0.472 and 0.472 (on a raw scale) and an alpha of 0.05.


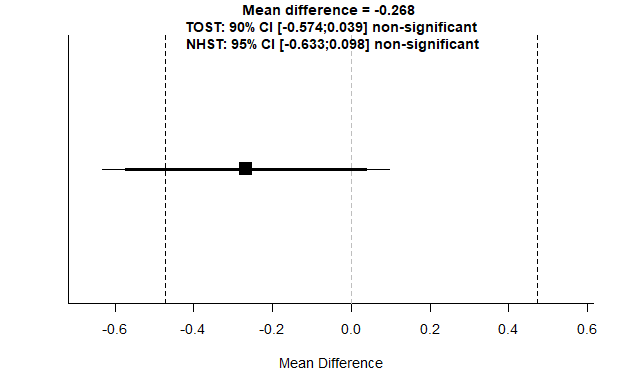


Figure S5c. Observed mean difference in likelihood to get external shading between parents and non-parents (in raw scale units), the equivalence bounds (also in raw scores), and the 90% and 95% CIs.
