# Supplementary material for: The role of parenthood in worry about overheating in homes in the UK and the US and implications for energy use: An online survey study
Source: PLoS One. 2022 Dec 1;17(12):e0277286. doi: 10.1371/journal.pone.0277286 (PMC9714918; doi:10.1371/journal.pone.0277286)
Supplement: S5 Appendix — (PDF) [file pone.0277286.s006.pdf]

S6 Appendix. Surveys copies

Two surveys have been developed, one for the US and one for the UK. The only differences between the surveys are different categories for tenure as socially rented is not applicable in the US and the questions on air-conditioning uptake and electric fans differ as specified in the hypotheses.

Overheating_UK_Parent

If you would like a copy of this information sheet, please print it from the screen.

Title of Study: The perception of overheating in UK homes
Department: UCL Energy Institute
Name and Contact Details of the Researchers:
Dr Gesche Huebner g.huebner@ucl.ac.uk

You are being invited to take part in a research project being conducted by the UCL Energy Institute and the UCL Institute for Environmental Design &Engineering.

Before you decide to take part, it is important for you to understand why the research is being done and what participation will involve. This research has been approved by the appropriate ethical board.

Participation is entirely voluntary. Please take time to read the following information carefully and discuss it with others if you wish. Email us if there is anything that is not clear or if you would like more information. Take time to decide whether or not you wish to take part.

Thank you for reading this.

1. What is the project's purpose?
This project aims at understanding to what extent UK homes get too warm in the summer and how this perception varies across different parts of the population; for example, depending on gender, age, household composition or type of home.

2. Why have I been chosen?
You have been invited because you are a member of Prolific's participant panel and an adult between 25-44 years living in the UK.

3. Do I have to take part?
It is up to you to decide whether or not to take part. You can withdraw at any time without giving a reason. If you decide to withdraw, any data you have provided will be withdrawn and you will not receive your payment.

4. What will happen to me if I take part?
You will be asked some questions about yourself and your household; the dwelling you live in; if your home gets too hot in summer, how you feel about that and what actions against overheating you take, if any.
It is expected that the study will last around 12 minutes.

5. What are the possible disadvantages and risks of taking part?
There are no risks associated with this study. However, if for any reason you feel uncomfortable during the study, please feel free to terminate the study. For those questions that you might perceive as more personal, e.g. such as about your employment status, there is always the option of not answering the question and stating “Prefer not to say”.

6. What are the possible benefits of taking part?
If you complete the study, you will be paid; the hourly rate is £7.50; hence, for 12 minutes, the pay is £1.50. Your involvement in this study will help further our understanding of overheating in the UK. If you are interested in the findings of the study, feel free to email g.huebner@ucl.ac.uk and you will receive a description of the main aggregated findings after a few months.

7. What if something goes wrong?
If you have any questions about the research, please contact g.huebner@ucl.ac.uk; or t.oreszczyn@ucl.ac.uk or the Department’s Director of Ethics (Michelle Shipworth; m.shipworth@ucl.ac.uk)

However, if you feel your complaint has not been handled to your satisfaction, you can contact the Chair of the UCL Research Ethics Committee –ethics@ucl.ac.uk

8. Will my taking part in this project be kept confidential?
All the information that we collect about you during the course of the research will be kept strictly confidential. You will not be able to be identified in any ensuing reports or publications. Researchers will only receive anonymous data.

9. What will happen to the results of the research project?
Results from this study will be presented at academic conferences, published in academic journals, and may also be presented to regulatory or industry bodies. As all data is anonymous, you will never be able to be identified as a participant. The data itself will be made publicly available on UCL’s data repository or another data repository so that other researchers can use it, too.

10. Who is organising and funding the research?
University College London (UCL) is organising the research.

Contact for further information
Please contact Gesche Huebner (g.huebner@ucl.ac.uk) if you would like any further information.

Thank you for reading this information sheet and for considering to take part in this research study.

Q1: Title of Study: The perception of overheating in UK homes   Department: UCL Energy InstituteName and Contact Details of the Researchers:
Dr Gesche Huebner g.huebner@ucl.ac.uk (main point of contact)
Professor Tadj Oreszczyn t.oreszczyn@ucl.ac.ukThis study has been approved by the UCL Institute for Environmental Design and Engineering Ethics Lead Thank you for considering taking part in this research.Please consent to all the elements as detailed below by ticking the boxes.

- ☐ I confirm that I have read and understood the Information Sheet for the above study.
- ☐ I understand that I will be able to withdraw my data up until the end of the survey by terminating the survey.
- ☐ I consent to participate in the study. I understand that no personal information will be collected during this study.
- ☐ I understand that my information may be subject to review by responsible individuals from the University for monitoring and audit purposes.
- ☐ I understand that my participation is voluntary and that I am free to withdraw at any time without giving a reason.
- ☐ I understand the potential risks of participating.
- ☐ I understand the direct/indirect benefits of participating.
- ☐ I understand that I will be paid £1.50 for completion of this study.
- ☐ I agree that my anonymised research data may be used by others for future research.
- ☐ I confirm that I understand why I have been invited to participate as detailed in the Information Sheet.
- ☐ I am aware of who I should contact if I wish to lodge a complaint.
- ☐ I voluntarily agree to take part in this study.

Q2: Please enter your Prolific ID.

Q3: What type of accommodation do you live in?PLEASE TICK ONE ANSWER ONLY.

- ○ A detached house / bungalow
- ○ A semi-detached house
- ○ A terraced house (including end terrace)
- ○ A purpose-built flat
- ○ A flat in a converted house
- ○ Other

If you have chosen "other", please specify:

Q4: Do you (or your household) own or rent this accommodation?PLEASE TICK ONE ANSWER ONLY.

- ◯ Own outright
- ◯ Own with a mortgage / loan
- ◯ Rent it privately
- ◯ Rent it from council (Local authority) or housing association
- ◯ Live here rent-free
- ◯ Other

If you have chosen "other", please specify:

Q5: Which of the following best describes the place where you now live? PLEASE TICK ONE ANSWER ONLY.

- ◯ A large city
- ◯ A suburb near a large city
- ◯ A small city or town
- ◯ A rural area

Q6: How many rooms are available for use only by this household?Do NOT count:• bathrooms• toilets• halls or landings• rooms that can only be used for storage such as cupboardsCount all other rooms, for example:• kitchens• living rooms• utility rooms• bedrooms• studies• conservatoriesIf two rooms have been converted into one, count them as one room.WRITE IN NUMBER BELOW.

Q7: How many of these rooms are bedrooms? Include all rooms built or converted for use as bedrooms, even if they are not currently used as bedrooms.WRITE IN NUMBER BELOW.

Q8: Approximately when do you think your accommodation was built?PLEASE TICK ONE ANSWER ONLY.

- ◯ Before 1900
- ◯ 1900 to 1929
- ◯ 1930 to 1949
- ◯ 1950 to 1975
- ◯ 1976 to 1990
- ◯ 1991 to 2002
- ◯ 2003 onwards
- ◯ Don't know

Q9: Including you, how many household members are there in each of the following age groups in your household?PLEASE WRITE IN NUMBERS BELOW FOR EACH (if there is nobody in a category, you can leave it blank).Please also indicate if you have parental responsibility for any household member in that category.

	How many?	Tick if you have parental responsibility for any household member in this age category
0 years		☐
1- 2 years		☐
3-5 years		☐
6-10 years		☐
11-17 years		☐
18-24 years		
25-44 years		
45-64		
65-74 years		
75-84 years		
85 years and more		

Q10: During this summer (2021), is there someone usually at home on weekdays?PLEASE TICK ONE ANSWER ONLY.

○ Yes, all weekdays. ○ Yes, some weekdays. ○ No.

Q11: Which of the following best describes how you think of yourself?PLEASE TICK ONE ANSWER ONLY.

◯ Male ◯ Female ◯ In some other way ◯ Prefer not to say

Q12: Which age category do you fall into?PLEASE TICK ONE ANSWER ONLY.

◯ 25-29 ◯ 30-34 ◯ 35-39 ◯ 40-44

Q13: Are you currently pregnant? If your sex is MALE, please choose "not applicable". PLEASE TICK ONE ANSWER ONLY.

◯ Yes ◯ No ◯ Don't know ◯ Not applicable ◯ Prefer not to say

Q14: Which best describes your current employment situation? PLEASE TICK ONE ANSWER ONLY.

◯ Working (paid or unpaid): 30 hours a week or more
◯ Working (paid or unpaid): less than 30 hours a week
◯ On maternity / paternity / shared parental / adoption leave
◯ Unemployed
◯ Student
◯ Not working because of longstanding disability or illness
◯ Not working for other reasons
◯ Prefer not to say
◯ Other

If you have chosen "other", please specify:

[ ]

Q15: How is your health in general? Is it …PLEASE TICK ONE ANSWER ONLY.

◯ Very good ◯ Good ◯ Fair ◯ Bad ◯ Very bad
◯ Prefer not to say

Q16: How often do you worry?PLEASE TICK ONE ANSWER ONLY.

◯ 0 (Never) ◯ 1 ◯ 2 ◯ 3 ◯ 4
◯ 5 ◯ 6 ◯ 7 ◯ 8 ◯ 9
◯ 10 (Continously) ◯ Prefer not to say

Q17: How much is worry a problem for you? PLEASE TICK ONE ANSWER ONLY.

- ◯ 0 (Not at all)
- ◯ 1
- ◯ 2
- ◯ 3
- ◯ 4
- ◯ 5
- ◯ 6
- ◯ 7
- ◯ 8
- ◯ 9
- ◯ 10 (Very much)
- ◯ Prefer not to say

Q18: To what extent would you call yourself a worrier?PLEASE TICK ONE ANSWER ONLY.

- ◯ 0 (Not at all)
- ◯ 1
- ◯ 2
- ◯ 3
- ◯ 4
- ◯ 5
- ◯ 6
- ◯ 7
- ◯ 8
- ◯ 9
- ◯ 10 (Very much)
- ◯ Prefer not to say

Q19: Overall, how satisfied are you with your life nowadays? PLEASE TICK ONE ANSWER ONLY.

- ◯ 1 (Not at all)
- ◯ 2
- ◯ 3
- ◯ 4
- ◯ 5
- ◯ 6
- ◯ 7
- ◯ 8
- ◯ 9
- ◯ 10 (Completely)
- ◯ Prefer not to say

Q20: During a typical summer (June to August), does your home overheat, i.e. does it get too hot? PLEASE TICK ONE ANSWER ONLY.

- ◯ Yes, in one room.
- ◯ Yes, in multiple rooms but not all rooms.
- ◯ Yes, in all rooms.
- ◯ No.

Q21: During a heatwave, does your home overheat, i.e. does it get too hot? PLEASE TICK ONE ANSWER ONLY.

- ◯ Yes, in one room.
- ◯ Yes, in multiple rooms but not all rooms.
- ◯ Yes, in all rooms.
- ◯ No.

Note: if you have NOT answered/chosen item [1, 2, 3] in question 20 OR answered/chosen item [1, 2, 3] in question 21, skip the following question

Q22: Please say which of these things, if any, you or your household sometimes do to when your home overheats during a typical summer and during a heatwave?PLEASE TICK ALL THAT APPLY.

	Typical summer day	During a heatwave
Open windows during the day to keep cool	☐	☐
Open windows at night to keep cool	☐	☐

Open external doors to keep cool	☐	☐
Open internal doors to keep cool (between rooms or internal spaces like shared hallways)	☐	☐
Use air conditioning	☐	☐
Use a portable electric fan to keep cool	☐	☐
Use a room-installed electric fan to keep cool	☐	☐
Use extractor fans to keep cool (per room or for the whole house)	☐	☐
Close blinds / curtains / shutters on the inside of windows or doors to keep cool	☐	☐
Close blinds / shutters on the outside of windows or doors to keep cool	☐	☐
Wear light clothes	☐	☐
Use light bedding	☐	☐
Have cold drinks to cool down	☐	☐
Have a bath or shower to cool off	☐	☐
Have a rest	☐	☐
Avoid using certain rooms in the home	☐	☐
Go outside	☐	☐
Go to a cooler building, away from the home	☐	☐
Other (please specify below)	☐	☐

Q23: Please judge the following items on overheating on a scale of from 1=never or rarely to 4= continously / almost always. PLEASE TICK ONE ANSWER PER ROW.

	1 (never / rarely)	2	3	4 (almost always / continously)	Prefer not to say
During this summer, how often have you thought about your home overheating?	○	○	○	○	○
During this summer, has thinking about your home overheating affected your mood?	○	○	○	○	○
During this summer, has thinking about the possibility of your home overheating affected your capacity to perform your “everyday activities”?	○	○	○	○	○
How often do you worry about the possibility of your home overheating?	○	○	○	○	○
Is being worried about your home overheating an important problem for you?	○	○	○	○	○

To what degree does the possibility of your home overheating worry you?	○	○	○	○	○

Note: if you have NOT answered/chosen at least one of the following items: [(3, 2), (4, 2), (5, 2), (3, 3), (4, 3), (5, 3), (3, 4), (4, 4), (5, 4), (3, 5), (4, 5), (5, 5), (3, 6), (4, 6), (5, 6)] in question 23, skip the following question

Q24: Please indicate why you are concerned about overheating in your home. If you are concerned for someone else, please explain for whom (e.g "my partner", "my child", "my grandparents"). Please only consider those living in your household. PLEASE TICK ALL THAT APPLY.

	Tick all that apply	Explain who are / is the "other(s)"
Negative health impacts (myself)	☐	
Negative health impacts (others) - – please specify for whom	☐	▭
Sleep disruption (myself)	☐	
Sleep disruption (others)	☐	▭
Feeling uncomfortable (myself)	☐	
Feeling uncomfortable (others)	☐	▭
Reduced productivity (e.g. less able to get work done) (myself)	☐	
Reduced productivity (e.g. less able to get work done) (others))	☐	▭
Other reasons (please specify)	☐	▭
Prefer not to say	☐	

Q25: Are there ever times when you would like to open a window or door to keep cool, but you don’t do it for one of these reasons? PLEASE TICK ALL THAT APPLY.

- ☐ No, this never happens.
- ☐ Yes – because of noise outside
- ☐ Yes – because of pollution outside
- ☐ Yes – because of weather conditions outdoors (e.g. wind, rain)
- ☐ Yes – because of concerns about security
- ☐ Yes – because of concerns about safety (e.g. to prevent children falling out)
- ☐ Yes – to keep pets in
- ☐ Yes – to keep animals/insects/pests out
- ☐ Yes – because it is difficult to open a window or keep it open the desired amount
- ☐ Yes – window cannot be opened at all (e.g. sealed, locked or faulty)
- ☐ Other, please specify

If you have chosen "other", please specify:

Q26: In the next three years, how likely are you to get an electric fan / additional electric fans for your home? (imagine you stay in the same home as now)PLEASE TICK ONE ANSWER ONLY.

- ○ 1 (Very unlikely)
- ○ 2
- ○ 3
- ○ 4
- ○ 5
- ○ 6
- ○ 7 (Very likely)

Note: if you have NOT answered/chosen item [1, 2] in question 4, skip the following question

Q27: In the next three years, how likely are you to get external shading for your home, e.g. external shutters, an awning or a tree? (imagine you stay in the same home as now)

- ○ 1 (Very unlikely)
- ○ 2
- ○ 3
- ○ 4
- ○ 5
- ○ 6
- ○ 7 (Very likely)

Q28: In the next three years, how likely are you to get an air conditioning system (portable or fixed) for your home? (imagine you stay in the same home as now)PLEASE TICK ONE ANSWER ONLY.

- ○ 1 (Very unlikely)
- ○ 2
- ○ 3
- ○ 4
- ○ 5
- ○ 6
- ○ 7 (Very likely)
- ○ Not applicable, already have it

Thank you so **much for participating in this study! Your answer will help to contribute to important research on overheating.**

If you are interested in the topic of overheating and heatwaves, you might find the following links of interest:
https://www.arcc-network.org.uk/extremes/overheating/overheating-in-homes-practical-advice/
https://www.nhs.uk/live-well/healthy-body/heatwave-how-to-cope-in-hot-weather/
https://youtu.be/oxijKUm5odM

Please note that this are external links and we don't have control over the content.
Please copy the completion code pasted below. Please make sure you click "Finish" BEFORE leaving the survey page.
COMPLETION CODE: 5118F007

Copy of Overheating_US_Parent

If you would like a copy of this information sheet, please print it from the screen.

Title of Study: The perception of overheating in US homes
Department: UCL Energy Institute
Name and Contact Details of the Researchers:
Dr Gesche Huebner g.huebner@ucl.ac.uk

You are being invited to take part in a research project being conducted by the UCL Energy Institute and the UCL Institute for Environmental Design &Engineering, London, United Kingdom.

Before you decide to take part, it is important for you to understand why the research is being done and what participation will involve. This research has been approved by the appropriate ethical board.

Participation is entirely voluntary. Please take time to read the following information carefully and discuss it with others if you wish. Email us if there is anything that is not clear or if you would like more information. Take time to decide whether or not you wish to take part.

Thank you for reading this.

1. What is the project's purpose?
This project aims at understanding to what extent US homes get too warm in the summer and how this perception varies across different parts of the population; for example, depending on gender, age, household composition or type of home.

2. Why have I been chosen?
You have been invited because you are a member of Prolific's participant panel and an adult between 25-44 years living in the US.

3. Do I have to take part?
It is up to you to decide whether or not to take part. You can withdraw at any time without giving a reason. If you decide to withdraw, any data you have provided will be withdrawn and you will not receive your payment.

4. What will happen to me if I take part?
You will be asked some questions about yourself and your household; the dwelling you live in; if your home gets too hot in summer, how you feel about that and what actions against overheating you take, if any.
It is expected that the study will last around 12 minutes.

5. What are the possible disadvantages and risks of taking part?
There are no risks associated with this study. However, if for any reason you feel uncomfortable during the study, please feel free to terminate the study. For those questions that you might perceive as more personal, e.g. such as about your employment status, there is always the option of not answering the question and stating “Prefer not to say”.

6. What are the possible benefits of taking part?
If you complete the study, you will be paid; the hourly rate is $9.50; hence, for 12 minutes, the pay is $1.90. Your involvement in this study will help further our understanding of overheating in the US. If you are interested in the findings of the study, feel free to email g.huebner@ucl.ac.uk and you will receive a description of the main aggregated findings after a few months.

7. What if something goes wrong?
If you have any questions about the research, please contact g.huebner@ucl.ac.uk; or t.oreszczyn@ucl.ac.uk or the Department’s Director of Ethics (Michelle Shipworth; m.shipworth@ucl.ac.uk)

However, if you feel your complaint has not been handled to your satisfaction, you can contact the Chair of the UCL Research Ethics Committee –ethics@ucl.ac.uk

8. Will my taking part in this project be kept confidential?
All the information that we collect about you during the course of the research will be kept strictly confidential. You will not be able to be identified in any ensuing reports or publications. Researchers will only receive anonymous data.

9. What will happen to the results of the research project?
Results from this study will be presented at academic conferences, published in academic journals, and may also be presented to regulatory or industry bodies. As all data is anonymous, you will never be able to be identified as a participant. The data itself will be made publicly available on UCL’s data repository or another data repository so that other researchers can use it, too.

10. Who is organising and funding the research?
University College London (UCL) is organising the research.

Contact for further information
Please contact Gesche Huebner (g.huebner@ucl.ac.uk) if you would like any further information.

Thank you for reading this information sheet and for considering to take part in this research study.

Q1: Title of Study: The perception of overheating in UK homes   Department: UCL Energy InstituteName and Contact Details of the Researchers:
Dr Gesche Huebner g.huebner@ucl.ac.uk (main point of contact)
Professor Tadj Oreszczyn t.oreszczyn@ucl.ac.ukThis study has been approved by the UCL Institute for Environmental Design and Engineering Ethics Lead Thank you for considering taking part in this research.Please consent to all the elements as detailed below by ticking the boxes.

- ☐ I confirm that I have read and understood the Information Sheet for the above study.
- ☐ I understand that I will be able to withdraw my data up until the end of the survey by terminating the survey.
- ☐ I consent to participate in the study. I understand that no personal information will be collected during this study.
- ☐ I understand that my information may be subject to review by responsible individuals from the University for monitoring and audit purposes.
- ☐ I understand that my participation is voluntary and that I am free to withdraw at any time without giving a reason.
- ☐ I understand the potential risks of participating.
- ☐ I understand the direct/indirect benefits of participating.
- ☐ I understand that I will be paid $1.90 for completion of this study.
- ☐ I agree that my anonymised research data may be used by others for future research.
- ☐ I confirm that I understand why I have been invited to participate as detailed in the Information Sheet.
- ☐ I am aware of who I should contact if I wish to lodge a complaint.
- ☐ I voluntarily agree to take part in this study.

Q2: Please enter your Prolific ID.

[ ]

Q3: What type of accommodation do you live in?PLEASE TICK ONE ANSWER ONLY.

- ◯ A detached house / bungalow
- ◯ A semi-detached house
- ◯ A terraced house (including end terrace)
- ◯ An apartment, flat
- ◯ A manufactured/mobile home
- ◯ Other

If you have chosen "other", please specify:

Q4: Do you (or your household) own or rent this accommodation?PLEASE TICK ONE ANSWER ONLY.

- ◯ Own outright
- ◯ Own with a mortgage / loan
- ◯ Rent it
- ◯ Live here rent-free
- ◯ Other

If you have chosen "other", please specify:

Q5: Which of the following best describes the place where you now live? PLEASE TICK ONE ANSWER ONLY.

- ◯ A large city
- ◯ A suburb near a large city
- ◯ A small city or town
- ◯ A rural area

Q6: How many rooms are available for use only by this household?Do NOT count:• bathrooms• toilets• halls or landings• rooms that can only be used for storage such as cupboardsCount all other rooms, for example:• kitchens• living rooms• utility rooms• bedrooms• studies• conservatoriesIf two rooms have been converted into one, count them as one room.WRITE IN NUMBER BELOW.

Q7: How many of these rooms are bedrooms? Include all rooms built or converted for use as bedrooms, even if they are not currently used as bedrooms.WRITE IN NUMBER BELOW.

Q8: Approximately when do you think your accommodation was built?PLEASE TICK ONE ANSWER ONLY.

- ◯ Before 1900
- ◯ 1900 to 1929
- ◯ 1930 to 1949
- ◯ 1950 to 1975
- ◯ 1976 to 1990
- ◯ 1991 to 2002
- ◯ 2003 onwards
- ◯ Don't know

Q9: Including you, how many household members are there in each of the following age groups in your household?PLEASE WRITE IN NUMBERS BELOW FOR EACH (if there is nobody in a category, you can leave it blank).Please also indicate if you have parental responsibility for any household member in that category.

	How many?	Tick if you have parental responsibility for any household member in this age category
0 years		☐
1- 2 years		☐
3-5 years		☐
6-10 years		☐
11-17 years		☐
18-24 years		
25-44 years		
45-64		
65-74 years		
75-84 years		
85 years and more		

Q10: During this summer (2021), is there someone usually at home on weekdays?PLEASE TICK ONE ANSWER ONLY.

◯ Yes, all weekdays. ◯ Yes, some weekdays. ◯ No.

Q11: Which of the following best describes how you think of yourself?PLEASE TICK ONE ANSWER ONLY.

◯ Male ◯ Female ◯ In some other way ◯ Prefer not to say

Q12: Which age category do you fall into?PLEASE TICK ONE ANSWER ONLY.

- ○ 25-29
- ○ 30-34
- ○ 35-39
- ○ 40-44

Q13: Are you currently pregnant? If your sex is MALE, please choose "not applicable". PLEASE TICK ONE ANSWER ONLY.

- ○ Yes
- ○ No
- ○ Don't know
- ○ Not applicable
- ○ Prefer not to say

Q14: Which best describes your current employment situation? PLEASE TICK ONE ANSWER ONLY.

- ○ Working (paid or unpaid): 30 hours a week or more
- ○ Working (paid or unpaid): less than 30 hours a week
- ○ On maternity / paternity / shared parental / adoption leave
- ○ Unemployed
- ○ Student
- ○ Not working because of longstanding disability or illness
- ○ Not working for other reasons
- ○ Prefer not to say
- ○ Other

If you have chosen "other", please specify:

Q15: How is your health in general? Is it …PLEASE TICK ONE ANSWER ONLY.

- ○ Very good
- ○ Good
- ○ Fair
- ○ Bad
- ○ Very bad
- ○ Prefer not to say

Q16: How often do you worry?PLEASE TICK ONE ANSWER ONLY.

- ○ 0 (Never)
- ○ 1
- ○ 2
- ○ 3
- ○ 4
- ○ 5
- ○ 6
- ○ 7
- ○ 8
- ○ 9
- ○ 10 (Continously)
- ○ Prefer not to say

Q17: How much is worry a problem for you? PLEASE TICK ONE ANSWER ONLY.

- ○ 0 (Not at all)
- ○ 1
- ○ 2
- ○ 3
- ○ 4
- ○ 5
- ○ 6
- ○ 7
- ○ 8
- ○ 9
- ○ 10 (Very much)
- ○ Prefer not to say

Q18: To what extent would you call yourself a worrier?PLEASE TICK ONE ANSWER ONLY.

- ◯ 0 (Not at all)
- ◯ 1
- ◯ 2
- ◯ 3
- ◯ 4
- ◯ 5
- ◯ 6
- ◯ 7
- ◯ 8
- ◯ 9
- ◯ 10 (Very much)
- ◯ Prefer not to say

Q19: Overall, how satisfied are you with your life nowadays? PLEASE TICK ONE ANSWER ONLY.

- ◯ 1 (Not at all)
- ◯ 2
- ◯ 3
- ◯ 4
- ◯ 5
- ◯ 6
- ◯ 7
- ◯ 8
- ◯ 9
- ◯ 10 (Completely)
- ◯ Prefer not to say

Q20: During a typical summer (June to August), does your home overheat, i.e. does it get too hot? PLEASE TICK ONE ANSWER ONLY.

- ◯ Yes, in one room.
- ◯ Yes, in multiple rooms but not all rooms.
- ◯ Yes, in all rooms.
- ◯ No.

Q21: During a heatwave, does your home overheat, i.e. does it get too hot? PLEASE TICK ONE ANSWER ONLY.

- ◯ Yes, in one room.
- ◯ Yes, in multiple rooms but not all rooms.
- ◯ Yes, in all rooms.
- ◯ No.

Note: if you have NOT answered/chosen item [1, 2, 3] in question 20 OR answered/chosen item [1, 2, 3] in question 21, skip the following question

Q22: Please say which of these things, if any, you or your household sometimes do to when your home overheats during a typical summer and during a heatwave?PLEASE TICK ALL THAT APPLY.

	Typical summer day	During a heatwave
Open windows during the day to keep cool	☐	☐
Open windows at night to keep cool	☐	☐
Open external doors to keep cool	☐	☐
Open internal doors to keep cool (between rooms or internal spaces like shared hallways)	☐	☐

Use air conditioning	☐	☐
Use a portable electric fan to keep cool	☐	☐
Use a room-installed electric fan to keep cool	☐	☐
Use extractor fans to keep cool (per room or for the whole house)	☐	☐
Close blinds / curtains / shutters on the inside of windows or doors to keep cool	☐	☐
Close blinds / shutters on the outside of windows or doors to keep cool	☐	☐
Wear light clothes	☐	☐
Use light bedding	☐	☐
Have cold drinks to cool down	☐	☐
Have a bath or shower to cool off	☐	☐
Have a rest	☐	☐
Avoid using certain rooms in the home	☐	☐
Go outside	☐	☐
Go to a cooler building, away from the home	☐	☐
Other (please specify below)	☐	☐

Q23: Please judge the following items on overheating on a scale of from 1=never or rarely to 4= continously / almost always. PLEASE TICK ONE ANSWER PER ROW.

	1 (never / rarely)	2	3	4 (almost always / continously)	Prefer not to say
During this summer, how often have you thought about your home overheating?	○	○	○	○	○
During this summer, has thinking about your home overheating affected your mood?	○	○	○	○	○
During this summer, has thinking about the possibility of your home overheating affected your capacity to perform your "everyday activities"?	○	○	○	○	○
How often do you worry about the possibility of your home overheating?	○	○	○	○	○
Is being worried about your home overheating an important problem for you?	○	○	○	○	○
To what degree does the possibility of your home overheating worry you?	○	○	○	○	○

Note: if you have NOT answered/chosen at least one of the following items: [(3, 2), (4, 2), (5, 2), (3, 3), (4, 3), (5, 3), (3, 4), (4, 4), (5, 4), (3, 5), (4, 5), (5, 5), (3, 6), (4, 6), (5, 6), (3, 7), (4, 7), (5, 7)] in question 23, skip the following question

Q24: Please indicate why you are concerned about overheating in your home. If you are concerned for someone else, please explain for whom (e.g "my partner", "my child", "my grandparents"). Please only consider those living in your household. PLEASE TICK ALL THAT APPLY.

	Tick all that apply	Explain who are / is the "other(s)"
Negative health impacts (myself)	☐	
Negative health impacts (others) - – please specify for whom	☐	▭
Sleep disruption (myself)	☐	
Sleep disruption (others)	☐	▭
Feeling uncomfortable (myself)	☐	
Feeling uncomfortable (others)	☐	▭
Reduced productivity (e.g. less able to get work done) (myself)	☐	
Reduced productivity (e.g. less able to get work done) (others))	☐	▭
Other reasons (please specify)	☐	▭
Prefer not to say	☐	

Note: if you have NOT answered/chosen item [1, 2] in question 4, skip the following question

Q25: In the next three years, how likely are you to get external shading for your home, e.g. external shutters, an awning or a tree? (imagine you stay in the same home as now)

○ 2 (Very unlikely) ○ 1 ○ 3 ○ 4 ○ 5
○ 6 ○ 7 (Very likely)

Q26: Do you currently have air-conditioning in your home (portable or fixed installed)? PLEASE TICK ONE ANSWER ONLY.

○ Yes. ○ No. ○ Don't know

Note: if you have NOT answered/chosen item [2, 3] in question 26, skip the following question

Q27: In the next three years, how likely are you to get an air conditioning system (portable or fixed) for your home? (imagine you stay in the same home as now)PLEASE TICK ONE ANSWER ONLY.

- ○ 1 (Very unlikely)
- ○ 2
- ○ 3
- ○ 4
- ○ 5
- ○ 6
- ○ 7 (Very likely)
- ○ Not applicable, already have it

Note: if you have NOT answered/chosen item [1] in question 26, skip the following question

Q28: In the next three years, how likely are you to get an additional air conditioning system (portable or fixed) for your home or replace the existing one with a higher performing one? (imagine you stay in the same home as now)PLEASE TICK ONE ANSWER ONLY.

- ○ 1 (Very unlikely)
- ○ 2
- ○ 3
- ○ 4
- ○ 5
- ○ 6
- ○ 7 (Very likely)

Q29: Are there ever times when you would like to open a window or door to keep cool, but you don’t do it for one of these reasons? PLEASE TICK ALL THAT APPLY.

- ☐ No, this never happens.
- ☐ Yes – because of noise outside
- ☐ Yes – because of pollution outside
- ☐ Yes – because of weather conditions outdoors (e.g. wind, rain)
- ☐ Yes – because of concerns about security
- ☐ Yes – because of concerns about safety (e.g. to prevent children falling out)
- ☐ Yes – to keep pets in
- ☐ Yes – to keep animals/insects/pests out
- ☐ Yes – because it is difficult to open a window or keep it open the desired amount
- ☐ Yes – window cannot be opened at all (e.g. sealed, locked or faulty)
- ☐ Other, please specify

If you have chosen "other", please specify:

Thank you so much for participating in this study! Your answer will help to contribute to important research on overheating.

If you are interested in the topic of overheating and heatwaves, you might find the following links of interest:

https://www.arcc-network.org.uk/extremes/overheating/overheating-in-homes-practical-advice/

https://www.nhs.uk/live-well/healthy-body/heatwave-how-to-cope-in-hot-weather/

https://youtu.be/oxijKUm5odM

Please note that this are external links and we don't have control over the content.

Please copy the completion code pasted below. Please make sure you click "Finish" BEFORE leaving the survey page.

COMPLETION CODE: 3B6A61DE
