# Supplementary material for: The role of parenthood in worry about overheating in homes in the UK and the US and implications for energy use: An online survey study
Source: PLoS One. 2022 Dec 1;17(12):e0277286. doi: 10.1371/journal.pone.0277286 (PMC9714918; doi:10.1371/journal.pone.0277286)
Supplement: S7 Appendix — (DOCX) [file pone.0277286.s008.docx]

**S8 Appendix. A checklist for reporting of tools that promote transparency, reproducibility, and quality of research**.

Citation:

Huebner, G. M., Fell, M. J., & Watson, N. E. (2021). Improving energy research practices: guidance for transparency, reproducibility and quality. *Buildings and Cities*, *2*(1), 1–20. https://doi.org/10.5334/bc.67

| **Tools** | **Check** | **Comments** |
| --- | --- | --- |
| ***Pre-registration*** |  |  |
| This study has pre-analysis plan. | Yes |  |
| *If yes* |  |  |
| URL |  | *https://osf.io/6395a/registrations* |
| Was it registered before data collection? | Yes |  |
| Does the paper mention and explain deviations from the PAP? | Yes | *Page 14 onward, labelled “Exploratory analysis”* |
| ***Reporting guidelines*** |  |  |
| This paper follows a reporting guideline. | Yes |  |
| *If yes* |  |  |
| Which one? |  | *STROBE (von Elm, E. et al. The Strengthening the Reporting of Observational Studies in Epidemiology (STROBE) statement: guidelines for reporting observational studies. Lancet 370, 1453–1457 (2007).)* |
| ***Open Data and Code*** |  |  |
| Data/code are publicly available | Yes, data and code |  |
| Does the paper make a statement on data and code availability? | Yes, on data and code | Data availability The data are uploaded on UCL’s figshare repository ( Code availability The code is uploaded in GMH’s Github page. |
| *If yes* |  |  |
| What is / are the link(s)? |  | <https://doi.org/10.5522/04/19294289.v1>; <https://github.com/Gesche-Huebner/Overheating> |
| Have steps been taken to ensure the data are FAIR? | Yes  No | *The data are but the documentation is not (PDF).* |
| Has meta-data been uploaded? | Yes |  |
| ***Preprints*** |  |  |
| Have you uploaded a preprint? | Yes |  |
| *If yes* |  |  |
| What is the link? |  | *10.31235/OSF.IO/3KVS7* |
| *If planned* |  |  |
| Which preprint server/location? |  |  |
